# Systematic Assessment of Immune Marker Variation in Type 1 Diabetes: A Prospective Longitudinal Study

**DOI:** 10.3389/fimmu.2019.02023

**Published:** 2019-09-13

**Authors:** Cate Speake, Henry T. Bahnson, Johnna D. Wesley, Nikole Perdue, David Friedrich, Minh N. Pham, Erinn Lanxon-Cookson, William W. Kwok, Birgit Sehested Hansen, Matthias von Herrath, Carla J. Greenbaum

**Affiliations:** ^1^Benaroya Research Institute at Virginia Mason, Seattle, WA, United States; ^2^Novo Nordisk Research Center Inc., Seattle, WA, United States; ^3^Clinical Development, Novo Nordisk A/S, Soborg, Denmark

**Keywords:** variability, immune assays, clinical trials, type 1 diabetes, insulin secretion

## Abstract

Immune analytes have been widely tested in efforts to understand the heterogeneity of disease progression, risk, and therapeutic responses in type 1 diabetes (T1D). The future clinical utility of such analytes as biomarkers depends on their technical and biological variability, as well as their correlation with clinical outcomes. To assess the variability of a panel of 91 immune analytes, we conducted a prospective study of adults with T1D (<3 years from diagnosis), at 9–10 visits over 1 year. Autoantibodies and frequencies of T-cell, natural killer cell, and myeloid subsets were evaluated; autoreactive T-cell frequencies and function were also measured. We calculated an intraclass correlation coefficient (ICC) for each marker, which is a relative measure of between- and within-subject variability. Of the 91 analytes tested, we identified 35 with high between- and low within-subject variability, indicating their potential ability to be used to stratify subjects. We also provide extensive data regarding technical variability for 64 of the 91 analytes. To pilot the concept that ICC can be used to identify analytes that reflect biological outcomes, the association between each immune analyte and C-peptide was also evaluated using partial least squares modeling. CD8 effector memory T-cell (CD8 EM) frequency exhibited a high ICC and a positive correlation with C-peptide, which was also seen in an independent dataset of recent-onset T1D subjects. More work is needed to better understand the mechanisms underlying this relationship. Here we find that there are a limited number of technically reproducible immune analytes that also have a high ICC. We propose the use of ICC to define within- and between-subject variability and measurement of technical variability for future biomarker identification studies. Employing such a method is critical for selection of analytes to be tested in the context of future clinical trials aiming to understand heterogeneity in disease progression and response to therapy.

## Introduction

The identification of highly effective disease-modifying therapies remains a critical gap in type 1 diabetes (T1D). While at least five different immunotherapies have been demonstrated to slow the decline in insulin secretion after diagnosis (1–5), not all who received active drug had a measurable therapeutic response. Improving the clinical success of immunotherapies is likely to require different strategies such as: use of combined or sequential therapies, selection of dosing frequency guided by changes in disease, and enrollment of cohorts most likely to respond. Biomarkers that predict clinical course and response to therapy may serve as stratification variables to understand disease and personalize intervention strategies. Given the promise of immunotherapy development in T1D, there is much interest in using immune analytes as biomarkers to predict clinical course and response to therapy. However, for such biomarkers to be implemented, it is critical to understand the variability of each marker between and within individuals as well as its relationship to clinical outcomes.

Many immune markers have been associated with different facets of T1D progression and response to therapy. For example, autoantibodies, measured using highly reproducible assays in multiple laboratories, have proven to be robust biomarkers. The presence of autoantibodies aids in the diagnosis of autoimmune diabetes in clinical settings, and, in asymptomatic individuals, autoantibodies are highly predictive of disease progression. However, to date, autoantibodies have not been useful in predicting the likelihood of therapeutic response. As T-cells are thought to play a role in T1D pathology, many aspects of T-cell frequency, function, and phenotype have been tested as potential biomarkers of disease or response to therapy. These features have been measured in both global T-cell populations (6–10) and antigen-specific subsets using a variety of methods (e.g., multimer/tetramer, ELISpot, and FluoroSpot assays) (11–14). Quantification, function, and phenotypes of B-cell, natural killer (NK) cell, and monocyte subsets have also been tested in T1D and proposed as biomarkers (15–19).

Previous efforts to evaluate biomarkers in T1D have focused on reliability—for example, technical reproducibility within or between laboratories (12, 20–22). Such reproducibility is a necessary but not sufficient criteria for clinical utility, as biomarkers should also be capable of explaining disease heterogeneity if they are to predict clinical outcomes. To our knowledge, evaluation of the utility of multiple immune measures on the same sample set, in a study designed specifically to evaluate variance within and between individuals, has not been previously performed.

Here, we sought to identify immune markers with specific characteristics that suggest they may provide insights into the heterogeneity of disease progression or response to therapy—namely, analytes with high between-subject and low within-subject variability. We also performed a pilot study to determine whether such analytes would demonstrate preliminary evidence of clinical relevance as measured by associations with insulin secretion. We designed a prospective longitudinal study to test the variance of a broad panel of 91 markers from 8 commonly used immune assays 9–10 times over a year, in a cohort of T1D subjects within 3 years of diagnosis (NCT01900834). We focused on three sources of variability: variability within subjects, variability between subjects, and technical assay variability. We rank-ordered each analyte tested according to intraclass correlation (ICC). For those with an acceptable ICC, we calculated a variable importance for the projection (VIP) score using a partial least squares (PLS) model. The ICC and VIP metrics served to quantify each biomarker's relative degree of within- and between-subject variability and its association with C-peptide levels (a measure of endogenous insulin secretion), respectively. Using this approach, we identified a panel of markers with low within-subject and high between-subject variability that associate with C-peptide levels shortly after T1D diagnosis. The association of the marker with the highest VIP score was then confirmed in an independent cohort of recent-onset T1D subjects [T1DAL, NCT00965458 (4)].

## Materials and Methods

### Study Design

#### Study Design—Prospective Observational Study

This was an observational study exploring the longitudinal variation of immune markers. This study was carried out in accordance with the recommendations of the ICH (ICH E6, 45CFR46) and FDA (21CFR sections 11, 50, 56, 312). The protocol was approved by the Benaroya Research Institute (BRI) Institutional Review Board (IRB) and all subjects gave written informed consent in accordance with the Declaration of Helsinki. Samples for this study were prospectively obtained from 15 healthy control subjects, 30 subjects with T1D, and 15 subjects with type 2 diabetes (T2D); all were enrolled in the BRI Immune Mediated Disease Registry and Repository Study. Key inclusion criteria were: for subjects with T1D, diagnosis <3 years, age 14–40 years, autoantibody positive; for subjects with T2D, diagnosis <15 years from enrollment, age 18–65 years; for healthy subjects, age 18–40 years. Medical history, concomitant illness and/or medications, and fasting status were collected for each subject at each visit. One T2D subject withdrew from the study after visit 2 and was replaced with a new subject per study protocol. Two other subjects (1 T2D, 1 control) withdrew from the study at later time-points; all available data are included for these two subjects. All available data are included in all analyses; outliers were neither identified nor removed from this study. Sample processing and preservation are described in detail in the Supplementary Methods.

While observational in nature, this clinical study was registered with clinicaltrials.gov (NCT01900834). The primary objective of the study was to assess longitudinal variation of immune markers in subjects with T1D over a 1 year period; subjects with T2D and healthy subjects served as controls. Primary and secondary endpoints specified variation of individual markers (CD8 T-cells, CD4 T-cells, insulin secretion, islet autoantibodies, and HbA1c). Each of these variables was assessed in the PLS modeling described below, using appropriate statistical methods as described.

#### Study Design—Replication

Technical reproducibility testing was conducted using 16 and 27 replicate aliquots from the same blood draw from up to six separate subjects, who were independent of those subjects enrolled in the natural history study (Data Sheet 2). Replicate tests were run at the beginning and end of each assay day. The range of the two replicate tests for each day was calculated, as well as the mean value of the two replicates. Upper and lower control limits for each analyte were calculated using the range as the variability estimate. The statistical control limits were calculated per-subject and represent three times the variability estimate divided by the square root of the sample size. Different subject samples were used for testing reproducibility of different assays and analytes, varying from 2 to 6 different subjects per analyte.

The natural history study was designed to understand variation in immune markers over time. Thus, the same markers were tested using samples from each of the 9–10 visits over 1 year. CD8 effector memory T-cell (CD8 EM) associations with insulin secretion were tested in two independent cohorts: the prospective cohort described above and the clinical trial cohort [Immune Tolerance Network (ITN) T1DAL cohort] described below. All assays were conducted by technicians blinded to study group.

#### Study Design—Confirmation Cohort (TIDAL Study)

We obtained clinical data from subjects enrolled in the ITN T1DAL trial (4, 23) via the TrialShare portal (24). Samples were originally collected in this multi-site study under the auspices of IRB-approved clinical trial protocols as described in the original trial publication (23). The Qdot-Multimer flow cytometry (QDM) assay was performed by Novo Nordisk Research Center Seattle, Inc. (NNRCSI) using protocols as described below and in Supplemental Materials, under the auspices of an independent collaboration between Novo Nordisk and the ITN. This independent study did not include other assays due to sample limitations.

#### Study Design—Control Samples for Assay Development and Implementation

For the ELISpot, FluoroSpot, and all flow cytometry assays except the HLA class II multimers, anonymous whole blood samples from subjects without diabetes, up to 100 mL, were purchased from Astarte Biologics (Bothell, WA, USA) and Puget Sound Blood Center (now BloodWorksNW, Seattle, WA, USA). Samples were processed to peripheral blood mononuclear cells (PBMC) and cryopreserved at the NNRCSI as described in Supplementary Methods.

### Clinical and Laboratory Assessments

#### Clinical Tests

Mixed meal tolerance tests (MMTT) were performed at study months 1, 5, and 9 for subjects with T1D using standard methods (25) (Supplementary Table 1). C-peptide, glucose, and HbA1c from all visits (including MMTT visits) were tested at the University of Washington's Northwest Lipid Metabolism and Diabetes Research Laboratories (Seattle, WA, USA). Serum samples for C-peptide were measured using a two-site immunoenzymatic assay on the Tosoh II 600 autoanalyzer (26). Complete blood count (CBC) measurements were performed on the day of each blood draw by Quest Diagnostics (Seattle, WA, USA).

#### HLA Typing

Presence of specific HLA DRB-1 and DQB1 genes were assessed by RT-PCR as previously described (27, 28). Initial low-resolution typing was performed to determine HLA-A serotype, as previously described (29). High-resolution typing was performed on the subset of subjects identified by low-resolution typing as HLA-A2+ (BloodWorks NW, Seattle, WA, USA) using isolated DNA and standard methods to further distinguish HLA-A2:01 donors from non-HLA-A2:01.

#### Autoantibody Measurements

Presence/absence of diabetes autoantibodies (GAD65, IA2, Insulin, and ZnT8) was determined in serum by the Barbara Davis Center Autoantibody/HLA Service Center using standardized radiobinding assays as previously described (30–32).

#### Qdot-Multimer Flow Cytometry (QDM)

Antigen-specific CD8+ T-cells were evaluated using a modified version of a previously described method (11). Peptide-HLA-A02:01 complexes were generated as described by Hadrup et al. (33) and in Supplementary Methods. pMHC and Qdot combinations are shown in Supplementary Table 2. After thawing, 1 ×10^6^ PBMC were incubated with Live/Dead Aqua dead cell stain (Molecular Probes, ThermoFisher, Waltham, MA, USA) in PBS for 30 min at room temperature. PBMC were then incubated with 500 nM dasatinib (LC Laboratories, Woburn, MA, USA) and multimers for 10 min at 37°C. Fluorescence minus one (FMO) controls for all multimers were incubated with PBS supplemented with 2% human AB serum (Life Technologies, ThermoFisher, Waltham, MA, USA) (FACS buffer) for 10 min at 37°C without multimers. Subsequently, cells were incubated in FACS buffer supplemented with additional fluorochrome-conjugated antibodies for 20 min at 4°C. Antibodies and cytometer filter set-up are listed in Supplementary Table 3. Data were acquired on an LSRII (BD Biosciences, San Jose, CA, USA) and analyzed using FlowJo software (Tree Star, Inc., Ashland, OR, USA). Samples from all subjects, regardless of HLA-A2 positivity, were subjected to the Qdot-Multimer testing as this enabled us to access the primary T cell flow cytometry data as well. Data for all subjects are presented here. The gating schema and representative flow cytometry data are shown in Supplementary Figures 1, 2. Detailed methods are included in the Supplementary Materials. Marker definitions and use are presented in Supplementary Table 4.

#### NK/Monocyte Flow Cytometry

After thawing, 1 ×10^6^ PBMC were incubated with Live/Dead Aqua dead cell stain (Molecular Probes, ThermoFisher, Waltham, MA, USA) in PBS for 30 min at room temperature. Cells were incubated in FACS buffer with fluorochrome-conjugated antibodies for 30 min at room temperature; all antibodies and cytometer filters are listed in Supplementary Table 5. Data were acquired on an LSRII (BD Biosciences, San Jose, CA, USA) and analyzed using FlowJo software (Tree Star, Inc; BD Biosciences, San Jose, CA, USA). Additional details provided in the Supplementary Methods. The gating schema and representative flow cytometry data are shown in Supplementary Figure 3, and marker definitions and use are shown in Supplementary Table 6.

#### HLA Class II Tetramer Assay

CD4 tetramer staining was conducted as previously described (34), on subjects that were HLA DR4+. In brief, cells were first treated with dasatinib (50 nM) for 10 min at 37°C before staining with a pool of PE-tetramer reagents (20 μg/mL) (Supplementary Table 7) for 2 h at room temperature. Cells were then treated with anti-PE beads (Miltenyi Biotec, Bergisch Gladbach, Germany) and enriched using a magnetic column. Cells were stained with a panel of antibodies, including anti-CD4, -CD45RO, -CD14 and -CD19, and further treated with Via-Probe (BD Biosciences, San Jose, CA, USA) before flow analysis. The gating schema and representative flow cytometry data are shown in Supplementary Figure 4.

#### ELISpot and FluoroSpot Assays for IL-10, IFN-γ, and IL-2 Detection

Pre-coated human IL-10 ELISpot kits and dual-color IFN-γ and IL-2 FluoroSpot kits were purchased from Mabtech AB (3430-4HPW-10 and FSP-0102-10, respectively; Stockholm, Sweden). Plates were washed and blocked for non-specific binding with PBS supplemented with 10% human HS-RPMI for 30 min at room temperature. Subsequently, thawed PBMC were added to each well at a concentration of 2.5 ×10^6^ cells/mL in 10% HS-RPMI for ELISpot and 2.0 ×10^6^ cells/mL for FluoroSpot assays. All peptide pools are described in Supplementary Table 8 and 0.5 μg PHA/mL media and 0.01% cell culture-grade DMSO were used as positive and negative stimuli, respectively. Cells were plated in triplicate and incubated at 37°C in 5% CO_2_ with humidity for 44–48 h. Plates were subsequently washed five times using a Biotek plate washer prior to addition of the detection antibody per the manufacturer's instructions (IL-10 ELISpot: 12-G8-biotin; IFN-γ/IL-2 FluoroSpot: 7-B6-1-FS-FITC; and MT8G10-biotin). Plates were incubated for 2 h at room temperature in the dark, washed to remove the detection antibody, and then developed per detailed description in the Supplementary Methods. After washing to remove development reagents, the ELISpot and FluoroSpot plates were allowed to air-dry fully (at least 60 h) in the dark. The number of spot-forming cells (SFCs) in the ELISpot and FluoroSpot assays were analyzed and counted using the AID multispot reader system (AID ELISpot Reader version 7.0 build 14790, AID GmbH, Strassberg, Germany). Detailed methods are included in the Supplementary Materials.

### Statistical Analysis

In order to quantify the proportion of immune analyte variation within and between subjects, the ICC was calculated using a linear mixed-effects model with a random effect for subject (35). In brief, ICC equals variance between subjects divided by the (variance between subjects + variance within subjects) and represents the proportion of the total variance that is between subjects. The code used to calculate ICC for this study is provided in the Supplementary Materials. The per-subject mean of C-peptide AUC, ng/mL/120 min was used as the PLS outcome, and markers with an ICC value above 70% were included in the PLS analysis. VIP was used for analyte selection (36). In the initial stage, the VIP was assessed in 35 analytes with an ICC value above 70%. Twenty-one markers with a VIP below 1.0 were dropped and 14 were retained. K-fold cross-validation with 7-folds was used to prevent overfitting and the minimum root mean predicted residual error sum of squares statistic was used to determine the number of factors (37). A final, one-factor PLS model was fit using all analytes above both the ICC threshold of 70% and the VIP threshold of 1.0. The code used to perform PLS analysis is also provided in the Supplementary Materials. Within each randomized arm of the confirmation cohort (T1DAL study) a PLS model was run without cross-validation using the per-subject mean values from the QDM assay as predictors and per-subject mean of the 4 h C-peptide AUC as the outcome. Because not all predictors with ICC above 70% in the original cohort were available in the confirmation cohort, we were not able to construct identical models. Instead, we used VIP to rank order the relative importance in predicting the C-peptide outcome and compared this metric across the cohorts.

## Results

### Study Design and Subject Recruitment

We designed a prospective clinical study to explore immune variability, enrolling 30 subjects within 3 years of T1D diagnosis, as well as 15 healthy control subjects and 15 subjects with T2D. Baseline subject characteristics are listed in Table 1. T1D and healthy subjects were well-matched for age. Slightly fewer female subjects were in the T1D group as compared to the T2D and healthy subject groups. As expected, T2D subjects were older and had higher BMI than both the T1D and the healthy subjects. All individuals underwent nine visits over a year, with visits separated by 1–3 months. Control and T1D subjects had one additional visit to assess variability over a week (Supplementary Table 1). Individuals in all groups maintained a high rate of participation throughout the study; only 2 of 60 subjects did not complete the study. In total, subjects attended 573 of 585 (97.9%) possible visits over the course of the study. CBC testing was done in a clinical laboratory using fresh samples. Samples for all other assays were cryopreserved on the day of collection. Assays were conducted in batch after clinical work was complete to minimize assay run-associated variability.

**Table 1:** Subject characteristics at enrollment.

	**Type 1 diabetes (*n* = 30)**	**Type 2 diabetes (*n* = 15)**	**Control (*n* = 15)**
Age, years; median (range)	24.5 (15–39)	51 (38–63)	28 (20–36)
Disease duration, years; median (range)	1.15 (0.2–2.8)	9.85 (2–19.5)	N/A
HbA1c, mmol/mol; median (range)	48 (26–121)	62 (49–80)	31 (26–34)
HbA1c, %; median (range)	6.5 (4.5–13.2)	7.8 (6.6–9.5)	5 (4.5–5.3)
C-peptide AUC, ng mL^−1^ 120 min^−1^; median (range)^a^	1.36 (<0.05–4.39)	N/A	N/A
BMI; median (range)	23.1 (19.7–27.8)	35.4 (25.1–45.0)	25.7 (19.8–31.5)
Female; *n* (%)	10 (33%)	8 (53%)	8 (53%)
Family history of type 1 diabetes; *n* (%)	11 (38%)	0 (0%)	1 (7%)
HLA-A*02; *n* (%)	17 (57%)	7 (47%)	7 (47%)
HLA-DR*0401; *n* (%)	14 (46%)	2 (13%)	2 (13%)

a *C-peptide data from first MMTT at visit 2 (1 month)*.

### The Variability of Immune Markers Differs Within and Across Assays

We performed several commonly used assays to assess the longitudinal variance of each analyte tested over 1 year and over 1 week. Markers measured included T1D-specific autoantibodies; T-cell, monocyte, and NK cell subset frequencies and activation status (using flow cytometry); frequencies of both CD4 and CD8 antigen-specific T-cells; antigen-specific T-cell function (using ELISpot/FluoroSpot); and CBC. Variability analyses incorporated data from all subjects, regardless of disease status.

**Figure 1:**
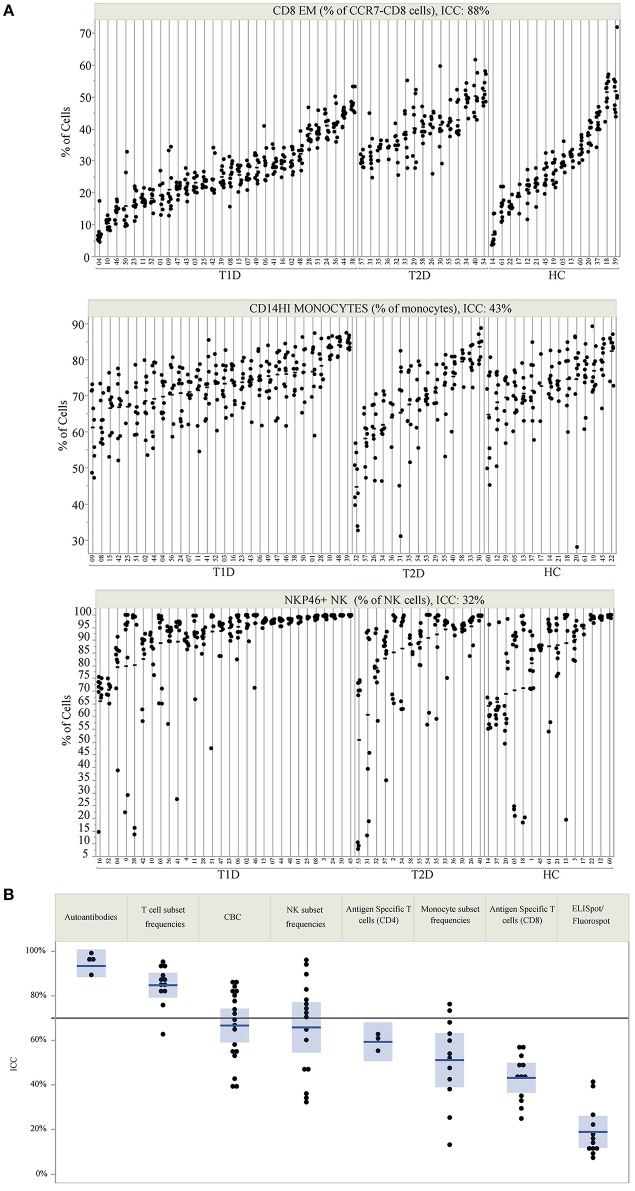
ICC identifies markers that are stable within an individual but vary between individuals. **(A)** Repeated assessments of CD8 EM, CD14HI Monocytes, and NKP46+ NK cells are shown for each subject. Subject IDs are listed on the X-axis, are divided by subject type, and are rank-ordered by the mean value of each immune marker within each subject type. The clustering of values by participant ID illustrates the relative amount of between- and within-subject variation. The total variation that is between subjects is quantified by the ICC, which is 88% for CD8 EM, 43% for CD14HI Monocytes, and 32% for NKP46+ NK cells. **(B)** The ICC for all measured markers is displayed by immune marker category. The reference line at 70% marks the threshold used to select markers for PLS modeling. ICC values are listed by marker in Table 2.

ICC was used to compare within- and between-subject variability of each analyte. Unlike coefficient of variation (CV) measurements, ICC performs well with values that approach 0. Since antigen-specific T-cells and other cell populations may be infrequent in the blood, ICC was a more appropriate measure of variability for this study. High ICC values represent analytes that were both longitudinally stable within subjects and biologically variable between subjects. For example, CD8 EM (defined as % of CD8+ cells that are CD4-CCR7-CD45RA-) have a high ICC of 0.88. This indicates that a given subject typically has similar CD8 EM frequencies throughout the year, as evidenced by the clustering of points for each subject [mean within-subject range 10.7% (95% CI: 9.04, 12.43)], but that individual subjects differ dramatically from each other (between-subject range 41.1%) (Figure 1A, top panel). In contrast, CD14 monocytes (Figure 1A, middle panel) and NKP46+ NK cells (Figure 1A, bottom panel) showed both less between-subject variability and more within-subject variability, reflected by lower ICC values of 0.41 and 0.32, respectively.

**Figure 2:**
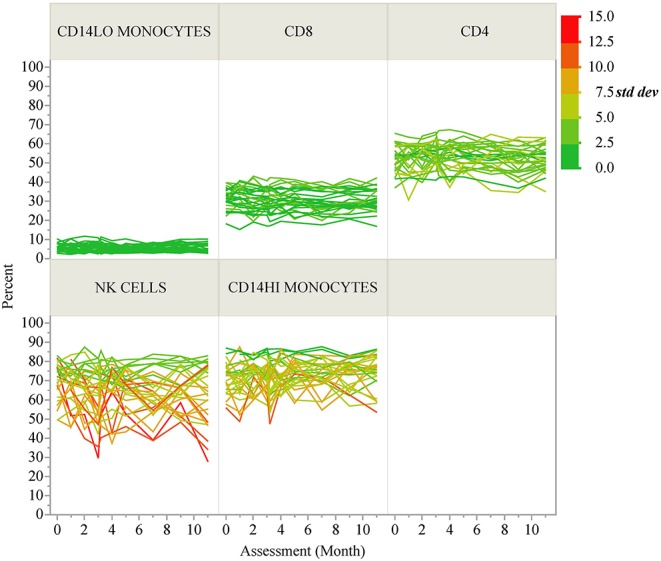
Longitudinal variability of selected immune cell populations. Spaghetti plots show the longitudinal variability of five selected immune populations. Individual lines correspond to T1D participants and the lines are colored according to the standard deviation of each participant's repeated assessments during the study.

ICC results are summarized in Figure 1B; each analyte is represented as a dot and the assay in which it was measured is displayed by column. ICC values for all analytes are listed in Table 2. ICCs were uniformly high for the four autoantibodies. In contrast, ICCs were uniformly low for analytes in the ELISpot/FluoroSpot functional cellular assays. Islet-specific CD4 and CD8 T-cells were also assessed using separate flow cytometry-based multimer assays. Moderate ICCs were achieved by both of these assays. Of note, total antigen-specific cells (summed across all antigen reactivities) were used for our analyses of both CD4 and CD8 multimer assays to increase cell frequency for each population. The frequency and ICC of non-antigen-specific T-cell subsets were also evaluated. CD4+CD8– and CD4-CD8+ populations were divided into: naïve (CD45RA+CCR7+), activated/Th1-like (CXCR3+), effector memory (CD45RA-CCR7-), central memory (CD45RA-CCR7+), and terminally differentiated effector memory (CD45RA+CCR7–). All populations are defined in Supplementary Tables 9–11. A separate flow cytometry panel was used to evaluate the frequency of monocyte and NK cell subsets. High ICCs were seen for nearly all T-cell subsets evaluated and for many of the monocyte and NK cell populations. To visualize the longitudinal change in cell frequency over the course of the year, the raw values for five example populations are presented in Figure 2. Analytes may have low/moderate ICC due to reduced between-subject variability, as exemplified by CD14lo monocytes, with ICC of 0.38, or due to high within-subject variability, represented by NK cells (%CD14–), with ICC of 0.6. Week to week ICC values are also listed in Supplementary Figure 5; these ICC tracked with the annual values.

**Table 2:** One year ICC estimates, rank-ordered according to ICC value and assay.

**Autoantibodies**		**T cell subset frequencies**		**CBC**		**NK subset frequencies**		**Antigen specific T cells (CD4)**		**Antigen specific T cells (CD8)**		**Monocyte subset frequencies**		**ELISpot/FluoroSpot**	
ZnT8	0.99	CD8N	0.94	MCV	0.86	CD2+ NK	0.96	CD4+TMR+ CD45RO+	0.62	AgSpc CD8 TEMRA CXCR3+	0.57	CD14Hi Mono HLA Class II+	0.76	IL-2-AR Pool	0.41
IA2	0.96	CD4N	0.94	MCH	0.86	CD57+ NK	0.94	CD4+TMR+	0.61	AgSpc CD8 TEMRA	0.57	CD14Lo Mono PDL1+	0.73	IFNG-QDM	0.4
GAD	0.96	CD8 TEMRA	0.93	Platelet Count	0.84	CD2+ NKHI	0.9	CD4+TMR+ CD45RO+	0.55	AgSpc CD8 TEMRA CXCR3+	0.53	CD14Hi Mono PDL1+	0.68	IFNG-AR Pool	0.27
IAA	0.89	CD4 CXCR3+	0.89	Abs. Lymphocytes	0.82	CD54+ NK	0.83			AgSpc CD8 TEMRA	0.49	CD 14Hi Mono CD2+	0.63	IFNG/IL-2-AR Pool	0.22
		CD8 EM	0.88	Hemoglobin	0.81	NKG2D+ NKHI	0.78			AgSpc CD8 EM	0.48	CD14Lo Monocytes CD2+	0.6	IL-2-INS Pool	0.18
		CD8 CM	0.87	Red Blood Cell Count	0.8	PDL1+ NKHI	0.76			AgSpc CD8 EM CXCR3+	0.44	CD14Hi Mono CD57+	0.54	IL-10-INS Pool	0.15
		CD8	0.85	Hematocrit	0.78	NKHI	0.73			AgSpc CD8 EM	0.44	CD14Lo Mono CD57+	0.52	IL-10-AR Pool	0.14
		CD4 CM	0.85	% Eosinophils	0.73	CD57+ NKHI	0.72			AgSpc CD8 EM CXCR3+	0.44	CD14Lo Mono HLA Class II+	0.48	IL-10-QDM	0.12
		CD4 TEMRA	0.82	Abs. Eosinophils	0.72	PDL1+ NK	0.7			AgSpc CD8 CM	0.35	CD14Hi Monocytes	0.43	IFNG-INS Pool	0.12
		CD4 EM	0.82	WBC Count	0.69	CD54+ NKHI	0.65			AgSpc CD8 CM CXCR3+	0.33	CD14Lo Monocytes	0.38	IL-2-QDM Pool	0.11
		CD8 CXCR3+	0.76	Abs. Monocytes	0.67	NK	0.6			AgSpc CD8 CM	0.3	CD14Lo Mono CD36+	0.25	IFNG/IL-2-INS Pool	0.09
		CD4	0.63	RDW	0.65	CD36+ NK	0.47			AgSpc CD8 CM CXCR3+	0.25	CD14Hi Mono CD36+	0.13	IFNG/IL-2-QDM	0.07
				% Monocytes	0.58	NKG2D+ NK	0.47								
				% Lymphocytes	0.56	NKP46+ NKHI	0.36								
				Abs. Neutrophils	0.54	CD36+ NKHI	0.34								
				% Neutrophils	0.53	NKP46+ NK	0.32								
				% Basophils	0.43										
				Abs. Basophils	0.4										
				MCHC	0.39										

We also compared the ICC values for each assay across disease groups (Supplementary Figure 6). In general, the ICC was highly similar regardless of disease status, and thus was very similar to the results presented in Figure 1, which show the overall result of all subjects in the study. An exception to this similarity is the islet autoantibody measurements, where the difference in ICC is a result of the healthy controls and T2D subjects having few to no positive autoantibodies and therefore little variability to explain at the subject level.

**Figure 3:**
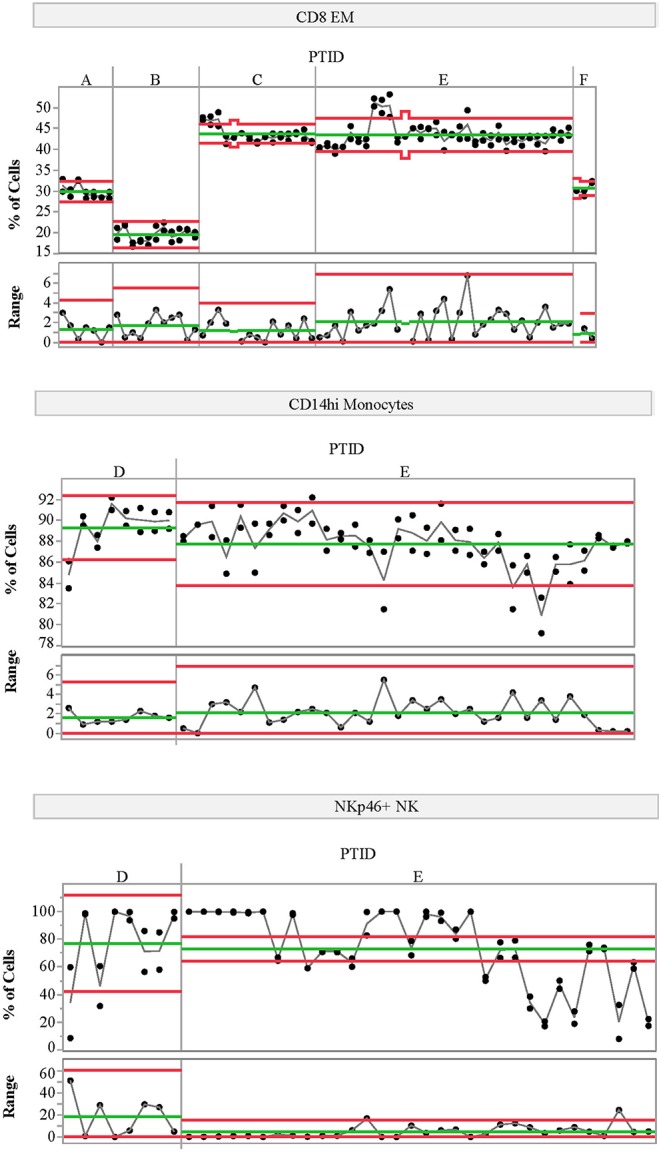
Variability charts of replicate control testing. Each chart displays the frequency of each population detected in two replicate aliquots from a single blood draw for a given control subject (top portion) measured on multiple experiment dates (x-axis). Replicate tests were run at the beginning and end of each day. The bottom portion of each control chart displays the range of the two replicate tests for each day. Subject IDs (PTID) are labeled A–F; these subjects are not the same as those included in the natural history study. Green lines represent the mean cell frequency for the two replicate measurements (top portion) and range (bottom portion) for each subject. The red lines represent the upper and lower control limits calculated using the range as the variability estimate. The statistical control limits are calculated per-subject and represent three times the variability estimate divided by the square root of the sample size.

We also assessed the technical variability of all analytes in the T-cell and NK/monocyte flow cytometry panels, as well as the CD8 antigen-specific cell populations and analytes included in the ELISpot/FluoroSpot assays (*n* = 64 total analytes). We compared results from 2 PBMC aliquots per day tested on multiple assay dates; all samples were processed from a single blood draw collected from up to six control subjects. These subjects were not participants in the clinical study described in Table 1. For some analytes, technical variability, as represented by the range of values detected within a subject over time, was low. For example, the maximum range within a subject for CD8 EM was <10% over 6 months of measurements (Figure 3, top panel). Other analytes such as CD14hi monocytes (Figure 3, middle panel) also had good technical variability (limited ranges on repeated measures from the same sample tested on different dates). The moderate ICC value (ICC = 0.43) for the CD14hi monocytes is therefore driven by limited variation between subjects as evidenced in Figure 1A. A contrasting example is the frequency of NK cells expressing NKP46. This population had poor technical variability (range of values on same samples tested over time and within an experiment between 20 and 30%; Figure 3, bottom panel) and showed a reduced ICC (ICC = 0.32). This increased technical variation leads to increased difficulty in understanding the relative contribution of within-subject and technical variability to low ICC. All available technical variability data are plotted and made available online at the following link (Data Sheet 2).

### Multivariate Modeling Identifies CD8 EM Association With C-Peptide in Subjects With T1D

Thirty-five analytes from five sets of assays had an annual ICC > 0.7 (Figure 1B), and were therefore included as candidates for multivariate modeling of insulin secretion assessed by C-peptide. Numerous studies have highlighted heterogeneity between individuals with respect to insulin secretion over time, and preservation of insulin secretion post-diagnosis is associated with reduced complications (38). The subjects enrolled in this study were diagnosed primarily as adults (Table 1); thus, their rate of fall in insulin secretion was, as expected, low over the course of the 1 year of follow-up (Supplementary Figure 7). For this reason, it was feasible to use mean insulin secretion over the year for each subject in the model. Similarly, because we selected only markers with a high ICC (low longitudinal variability), we also used the mean value of each immune marker in the model. This reduces the noise of all parameters in the model, improving statistical power.

**Figure 4:**
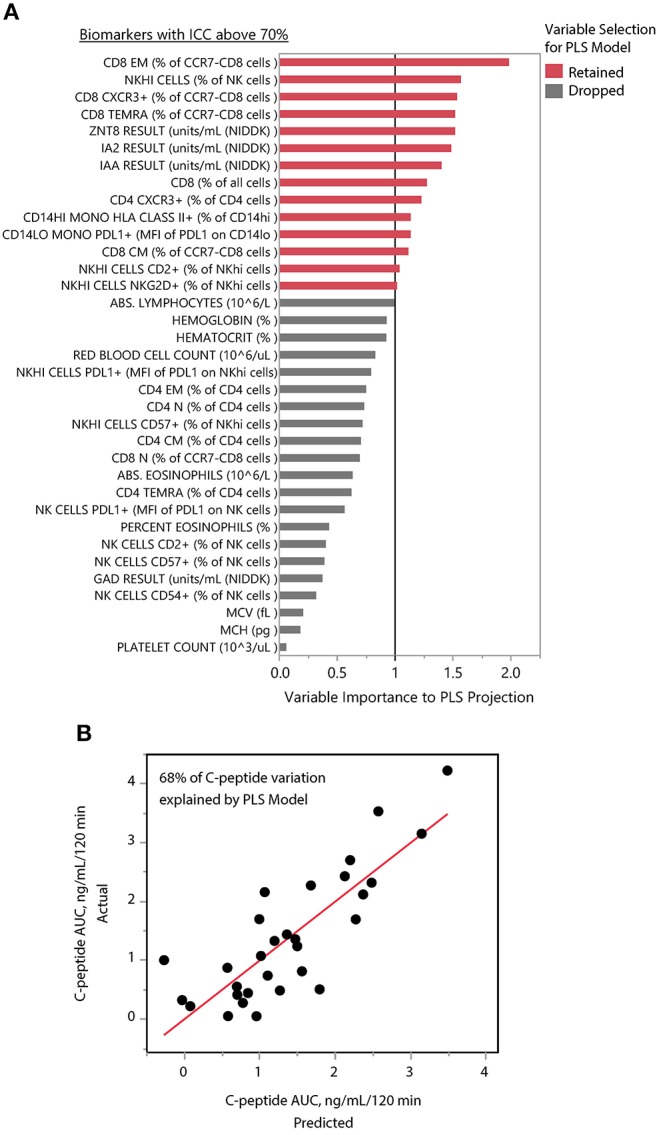
PLS identifies composite model associated with insulin secretion. **(A)** The mean levels of 35 immune markers with ICCs above 70% were used to model mean C-peptide over 1 year using PLS. VIP scores for 14 markers (red) had VIP scores above 1.0 and were retained in the PLS model. The other 21 markers (gray) were dropped from the multivariate model as their importance to the PLS projection was minimal. **(B)** The final model explained 68% of the variability of the mean C-peptide over 1 year using a 1-factor model created from weighted linear combinations of 14 markers. Y-axis indicates the actual C-peptide mean values for each subject; each subject is a dot. X-axis indicates the C-peptide values predicted by the PLS model.

**Figure 5:**
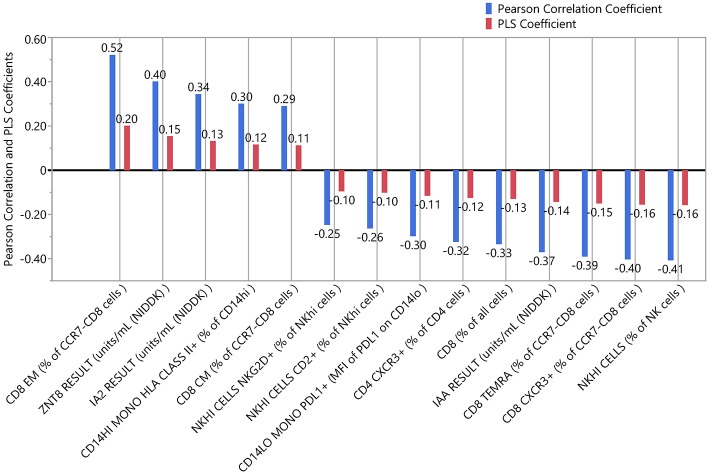
Independent and combined associations of each marker with insulin secretion. The standardized and scaled PLS coefficients (red) are multivariate adjusted associations between each marker and insulin secretion. Blue indicates the bivariate, unadjusted Pearson correlation coefficients for the same association. Coefficients above zero indicate a positive association; below zero indicates a negative association.

We applied PLS analysis to our dataset to select markers that were associated with C-peptide. PLS combines features of principal component analysis and multivariate linear regression to select variables associated with one or more outcomes. Importantly, PLS incorporates a variable selection criteria (VIP) that has been shown to be a reliable and parsimonious method to filter analytes/features before implementing PLS regression (39). Using a VIP threshold of 1.0, we selected a panel of 14 (out of 35) markers to evaluate their contribution to C-peptide variation (Figure 4A). CD8 EM had both the highest independent association with C-peptide and the highest PLS VIP score; the bivariate correlation between %CD8 EM and C-peptide was 0.52 (Rho *p*-value = 0.003). Independent association between any particular marker and C-peptide was limited, but, taken together, the PLS model explained 68% of the between-subject variability in C-peptide (Figure 4B). The 14 markers had varying individual associations with C-peptide, and were similar in magnitude and direction to simple bivariate Pearson correlation coefficients (Figure 5).

### C-Peptide Association and CD8 EM Stability in a Second Recent-Onset T1D Cohort

**Figure 6:**
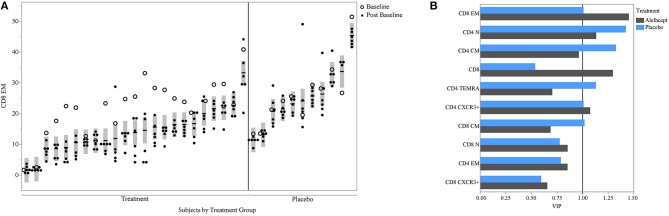
CD8 EM and relationship with insulin secretion in T1DAL cohort. **(A)** ICC of CD8 EM is similar in a second cohort (ICC = 75% overall and 82% after removing the baseline assessments in the alefacept group). Y-axis is frequency of CD8 EM; X-axis groups the repeated measures for each subject. The per-subject mean is marked with the horizontal line and the shaded bar represents the 95% confidence interval of the mean. Open circles indicate first visit (prior to alefacept in treatment arm); all other visits (closed circles) were post-baseline assessments. Figure is paneled by treatment group. **(B)** The variable importance measure (VIP) is displayed on the x-axis from the PLS model associating insulin secretion with T-cell markers for each treatment group.

PLS can help avoid overfitting with the use of cross-validation techniques. Therefore, we used k-fold cross-validation in our PLS analysis. We also aimed to determine whether our findings could be seen in a second cohort; for this, we used data from the T1DAL trial. T1DAL was a randomized placebo-controlled trial conducted in new-onset T1D subjects that aimed to delay C-peptide decline after T1D diagnosis by treating subjects with the LFA-3Ig fusion protein, alefacept (4). The full panel of assays used for our original study could not be conducted using samples from this trial. However, the antigen-specific CD8 assay and assessment of T-cell subset frequencies were tested against this sample set using the same QDM flow cytometry assay protocol, same operators, and same flow cytometers. As expected, CD8 EM levels dropped after alefacept treatment in the majority of subjects (as evident in the comparison between open circles representing the baseline time-point and closed circles representing post-baseline time-points, Figure 6A). Subjects still show similar within- and between-subject variability in this independent dataset, with an ICC value of 75% when the baseline value is included, or 82% when the value is removed from the ICC calculation. When PLS modeling was performed on this independent dataset, CD8 EM was again identified as the most informative subset in its association with mean C-peptide levels in the treatment arm, and was also above the PLS cut-off value in the small placebo arm (Figure 6B).

## Discussion

High ICC values, low technical variability, and association with biological measures of interest are desirable characteristics of immune markers when aiming to address heterogeneity of disease, either in the natural history of disease progression or response to therapy. This systematic evaluation of 91 putative biomarkers identified 35 with high ICC values. The combination of analytes selected by PLS modeling accounted for 68% of the variation in insulin secretion in our initial cohort. CD8 EM had the highest VIP score using PLS modeling, as well as the highest bivariate Pearson correlation with C-peptide. The high ICC value of CD8 EM cells and their association with insulin secretion was confirmed in a separate cohort. Not unexpectedly, in addition to CD8 EM, we also identified autoantibodies as another marker with a high ICC and some association with C-peptide. Autoantibodies against islet proteins are currently used as biomarkers to define risk of progression to T1D diagnosis. Autoantibodies were identified and validated through a community effort, with shared sample sets distributed to laboratories world-wide for assessments and a well-accepted qualification process for each laboratory (40, 41), assuring a high level of technical reproducibility.

Analytes with the lowest ICC in our study include many of the antigen-specific measures. One challenge in detecting antigen-specific cells is their low frequency in subjects with T1D, which may be partially attributable to under detection (42). We assessed the technical reproducibility of two antigen-specific measures, the antigen-specific CD8 T-cell assay and ELISpot/Fluorospot assays. Antigen-specific CD8 T-cells measured by flow cytometry had adequate technical reproducibility and consistently detected a very low frequency of antigen-specific cells in peripheral blood. This translated into low between-subject variability; measuring larger volumes of cells or looking in different populations could in future show different results. The ELISpot/FluoroSpot assay showed considerable technical variability as evidenced by high within-subject ranges during technical replicate testing. We did not test whether the use of frozen as compared to fresh samples contributed to the relatively low technical reproducibility seen here and in previous work (20). While other studies (43–45) have shown relatively similar results in fresh compared to frozen samples after background subtraction, this remains an open question in the field. Our data suggests that the antigen-specific analytes tested on frozen samples are unlikely to be useful in the context of understanding the natural history of disease. However, these assays could potentially be useful in the context of a clinical trial evaluating a therapy expected to increase the frequency, or modify the phenotype, of antigen-specific cells. In such a case, these assays could serve as a pharmacodynamic marker of drug administration or a marker of therapeutic efficacy.

While specific populations of CD8 EM have been identified as predictors of response to therapy (46) and C-peptide decline in children (47), total levels of CD8 EM have not been previously shown to be associated with C-peptide levels in the natural history setting and were not identified in the original assessment of the T1DAL study. This new finding may have been possible due to the specifics of our analysis method. Our discovery cohort included older subjects with relatively little decline in insulin secretion, and we selected analytes with a high ICC, enabling us to use mean insulin secretion and mean levels of each immune marker for our analyses. This reduced the within-subject variability for both insulin secretion and the immune markers, increasing the power to detect associations. Also, PLS works well in cases where multiple modest or noisy correlations may predict an outcome when combined; this likely applies to the T1D setting. One potential explanation for the association observed between insulin secretion and CD8 EM is an increased frequency of CD8 T-cells expressing an exhaustion phenotype in subjects with increased CD8 EM. Cells expressing an exhaustion phenotype have been associated with improved outcomes in T1D (46, 48). However, the frequency of these cells was not assessed in the context of this study. An alternate explanation can be found in the “full” immune system hypothesis (49), which postulates that higher total T-cell counts tend to prevent an increase in homeostatic T-cell proliferation. Preventing this proliferation may limit expansion of self-reactive T-cell populations. Of course, follow-up studies to explore the possible immunological and clinical implications of this association are needed to fully explain this intriguing association.

Low technical variability, high ICC values, and association with biological measures of interest are desirable characteristics of an analyte when aiming to address heterogeneity in disease, either in the natural history of disease progression or response to therapy. The known heterogeneity in disease progression and response to therapy in T1D and other autoimmune and inflammatory diseases suggests that a more personalized approach is needed for therapeutic selection and trial enrollment. The flow cytometry data generated here are particularly useful to understand and characterize the within- and between-subject variability of immune markers. Analytes with high ICC allow personalized ranges and deviations to be correlated with biological events (such as loss of insulin secretion) or therapeutic response. ICC, used in this context, measures the percent of variability in a sample that can be explained by the subject. It is, therefore, a metric quantifying the degree of marker personalization. Immune markers with high ICC should be more useful in personalizing therapies by blocking, stratifying, or serving as baseline covariates, thereby increasing statistical power and precision (50). Further studies, both retrospective and prospective, are needed to determine the utility of the markers tested here in T1D clinical trials, but the method described is generalizable to studies in other settings. Such studies should also ensure that feasibility of the biomarkers is assessed, including considerations such as cost, wide transferability to clinical laboratories, and ease of sample collection.

Through a systematic approach, we have identified multiple immune markers that are stable in an individual but vary within the population. While our discovery work was performed almost solely in adult subjects, we note that the ICC of CD8 EM was similar between our prospective cohort and the T1DAL study, which enrolled pediatric subjects, suggesting that the ICC findings here may be generalizable to the pediatric setting. It is not known whether these ICC results would differ if tested prior to clinical diagnosis of T1D, nor whether the within- and between-subject variation for any given analyte would apply equally well in other disease settings. As mentioned, additional follow-up will be required to understand the potential relationship between CD8 EM and insulin secretion. In summary, we propose that this approach can be applied to identify analytes worthy of consideration as biomarkers to dissect heterogeneity in T1D and predict response to therapy.

## Data Availability

The raw data supporting the conclusions of this manuscript will be made available by the authors, without undue reservation, to any qualified researcher. Technical replicate control plots of all 64 analytes are available online at (Data Sheet 2) and are oriented similarly as Figure 3. Each chart displays the frequency of a given population detected in two replicate aliquots from a single blood draw for each subject (top portion) measured on multiple experiment dates (x-axis). Replicate tests were run at the beginning and end of each day. The bottom portion of each control chart displays the range of the two replicate tests for each day. Subject IDs (PTID) are labeled A–F; multiple control subjects were tested for each immune marker. Green lines represent the mean cell frequency for the two replicate measurements (top portion) and range (bottom portion) for each subject. The red lines represent the upper and lower control limits calculated using the range as the variability estimate. The statistical control limits are calculated per-subject and represent three times the variability estimate divided by the square root of the sample size.

## Ethics Statement

This study was carried out in accordance with the recommendations of the ICH (ICH E6, 45CFR46) and FDA (21CFR sections 11, 50, 56, 312). The protocol was approved by the Benaroya Research Institute Institutional Review Board. All subjects gave written informed consent in accordance with the Declaration of Helsinki.

## Author Contributions

CG, MH, JW, and BS conceived of and designed the study. WK, JW, NP, EL-C, DF, MP, and CS collected data and conducted preliminary analysis. Data cleanup and curation was performed by CS, BS, JW, and HB. Formal statistical analysis was conducted by HB. CS and HB wrote the first draft of the manuscript. CS, HB, JW, BS, EL-C, DF, NP, and CG wrote and edited the manuscript. All authors approve of the content. CG is the guarantor of this work, she accepts full responsibility for the work and conduct of the study, had access to the data, and controlled the decision to publish.

### Conflict of Interest Statement

The study was funded by Novo Nordisk: the funder was involved in the study design, collection, analysis, interpretation of data, the writing of this article and the decision to submit it for publication. CG received research support from Janssen, Inc. in the preceding 12 months. JW, NP, DF, MP, EL-C, and MH received a salary from Novo Nordisk Research Center Seattle throughout the course of this work. BS received a salary from Novo Nordisk A/S throughout the course of this work. The remaining authors declare that the research was conducted in the absence of any commercial or financial relationships that could be construed as a potential conflict of interest.
