# Supplementary material for: Systematic Assessment of Immune Marker Variation in Type 1 Diabetes: A Prospective Longitudinal Study
Source: Front Immunol. 2019 Sep 13;10:2023. doi: 10.3389/fimmu.2019.02023 (PMC6753618; doi:10.3389/fimmu.2019.02023)
Supplement: Supplementary file 1 [file Data_Sheet_1.pdf]

## *Supplementary Material*

1 Supplementary Figures ....................................................................................................3

1.1 Supplementary Figure S1. Flow cytometry gating scheme used for the analyses of T-cell subsets, including multimer+ cells. ....................................................................................3

1.2 Supplementary Figure S2. Representative data for T-cell subset analyses. ..........................4

1.3 Supplementary Figure S3. Representative data for NK cell and monocyte subset analyses. ..5

1.4 Supplementary Figure S4. Flow cytometry gating scheme used for the analyses of antigen specific CD4+ T cells. ......................................................................................................6

1.5 Supplementary Figure S5. One year and one week ICC analyte estimates. ...........................7

1.6 Supplementary Figure S6. Mean and range of ICC values for all subjects compared to T1D, T2D, or controls..............................................................................................................8

1.7 Supplementary Figure S7. Little change in insulin secretion over 1 year in T1D cohort. ......9

2 Supplementary Tables ...................................................................................................10

2.1 Supplementary Table S1. Schedule of assessments for longitudinal biomarker study..........10

2.2 Supplementary Table S2. Multimer-Qdot combinations used for antigen-specific CD8 T-cell assay. 11

2.3 Supplementary Table S3. Summary of assay characteristics detailing antibody clones, vendors, LSRII laser and filters used for the QDM flow cytometry panel. ...................................12

2.4 Supplementary Table S4. QDM Phenotyping Marker Definitions, Excluding Multimers, and Use. 13

2.5 Supplementary Table S5. Summary of assay characteristics detailing antibody clones, vendors, LSRII laser and filters used for the NK/monocyte flow cytometry panel. .......................14

2.6 Supplementary Table S6. NK/Monocyte Panel Marker Description and Use. ....................15

2.7 Supplementary Table S7. Peptides used for antigen-specific CD4 T-cell assay. .................16

2.8 Supplementary Table S8. Peptide pools used for the ELISpot and FluoroSpot assays. ........17

2.9 Supplementary Table S9. NK/Monocyte Flow Cytometry Panel Population Definitions. ...18

2.10 Supplementary Table S10. Main T-cell Subsets Flow Cytometry Panel Population Definitions. ...........................................................................................................................19

2.11 Supplementary Table S11. QDM Flow Cytometry Panel Population Definitions, Multimer+ Subsets..................................................................................................................................20

3 Supplementary Data .....................................................................................................21

3.1 Data file S1. Technical replicate control plots of all 64 biomarkers hyperlink. ...................21

4 Supplementary Detailed Materials and Methods ............................................................21

4.1 Whole blood collection and cryopreservation .................................................................21

4.2 Thawing cryopreserved PBMC .......................................................................................22

4.3 Qdot-Multimer flow cytometry (QDM) ....................22
4.4 LSRII QC Procedure....................24
4.5 ELISpot and FluoroSpot assays for IL-10, IFN-γ, and IL-2 detection ....................25
4.6 Code for statistical analyses....................26

## 1 Supplementary Figures

### 1.1 Supplementary Figure S1. Flow cytometry gating scheme used for the analyses of T-cell subsets, including multimer+ cells.

**(A)** Illustration of the quantum dot background exclusion (QBE) gating strategy used. This is a basic gating strategy to exclude potential background signal introduced by Qdots to each multimer pair. **(B)** Gating strategy for identifying T-cell subsets.

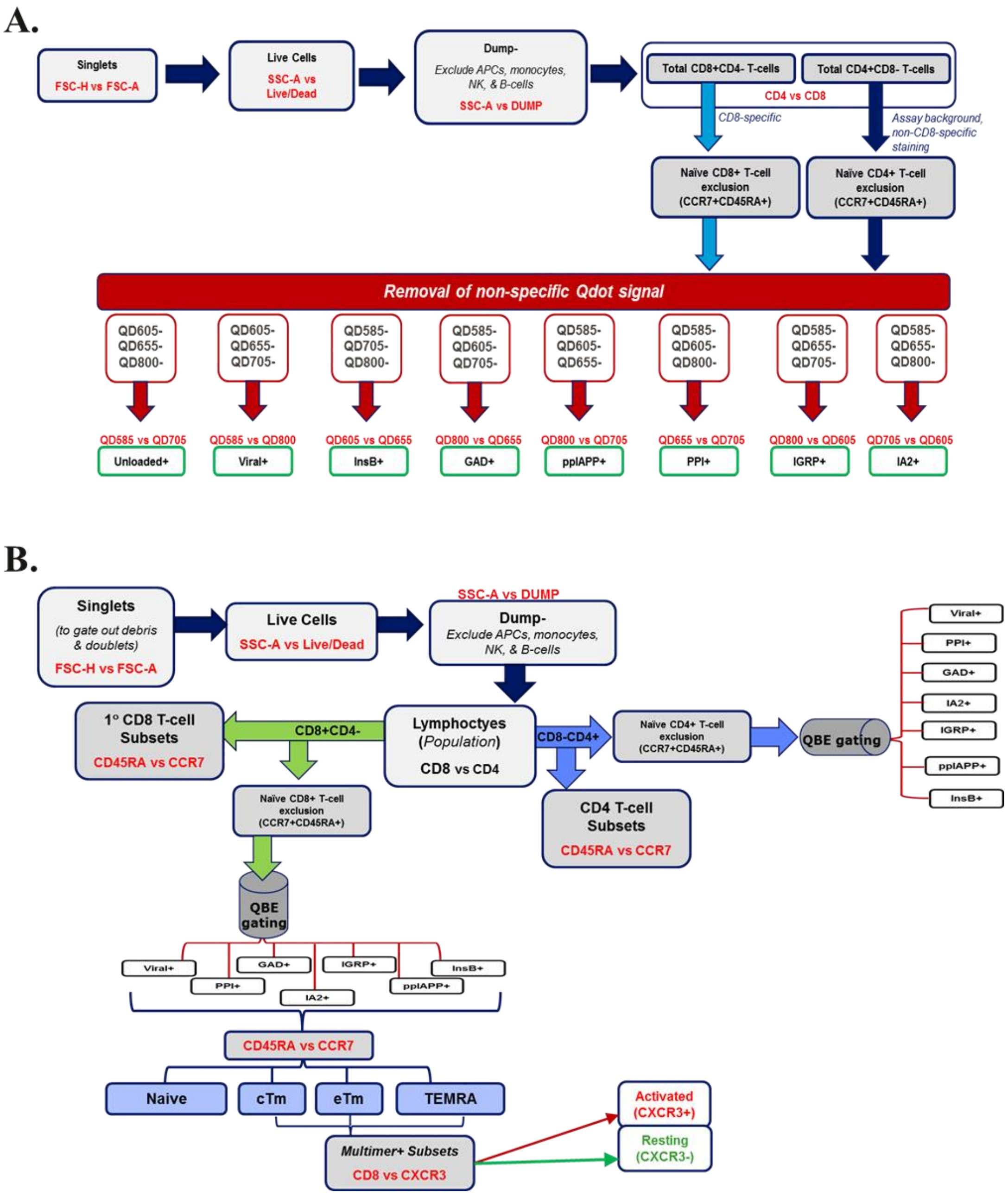

**1.2 Supplementary Figure S2. Representative data for T-cell subset analyses. (A)** Representative data showing gating scheme for all subsets, including QBE gating. **(B)** Representative multimer analyses.

**A.**

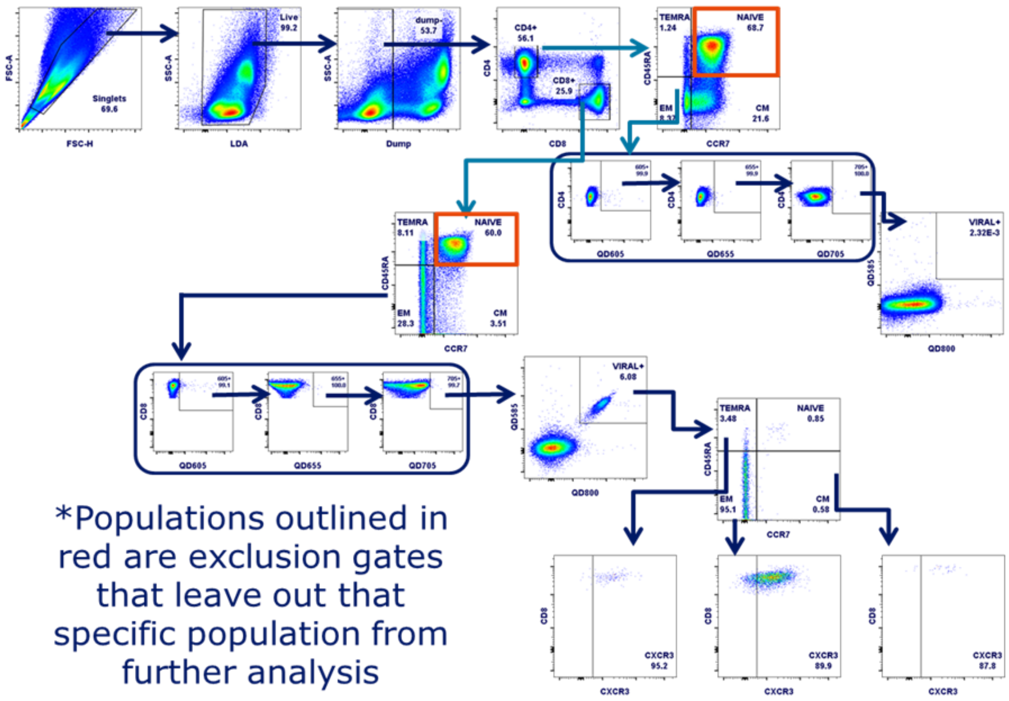


**B.**

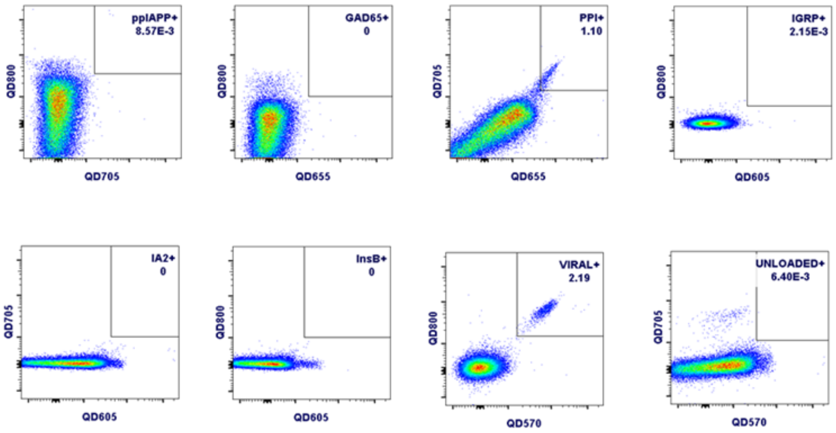

1.3 **Supplementary Figure S3. Representative data for NK cell and monocyte subset analyses. (A)** Example of monocyte subset gating. **(B)** Example of NK cell gating.

**A.**

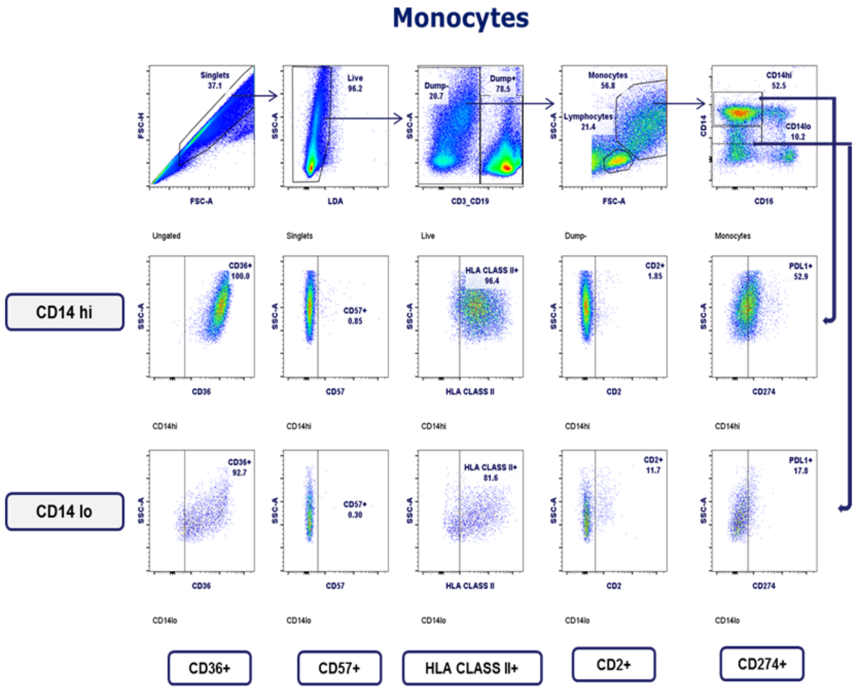


**B.**

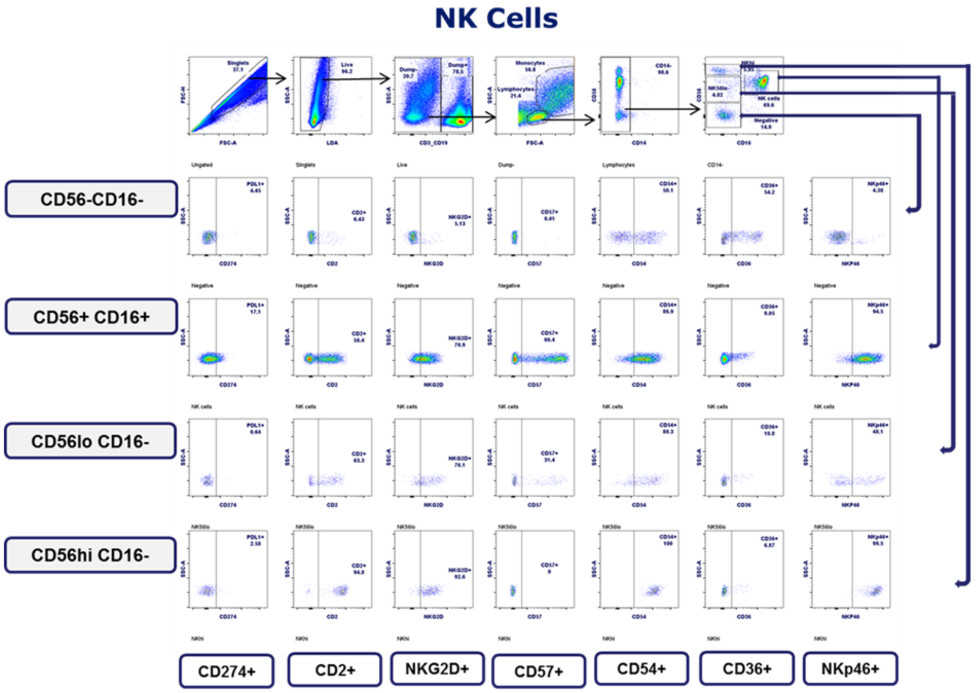

1.4 **Supplementary Figure S4. Flow cytometry gating scheme used for the analyses of antigen specific CD4+ T cells.** Lymphocyte populations were gated based on forward and side scatter. Viable cells that were CD14 and CD19 negative and CD4 positive were identified as CD4+ T cells. Memory and naïve antigen specific cells within CD4+ T cells were identified by using tetramers and CD45RO.

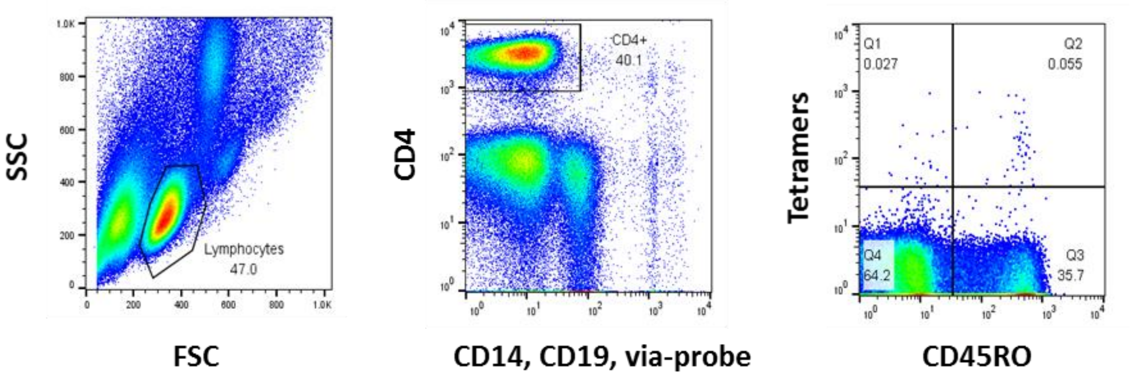

**1.5 Supplementary Figure S5. One year and one week ICC analyte estimates.** Each biomarker is rank-ordered according to ICC value within the corresponding assay. The left and right bar charts display the ICC estimates taken over one year of assessments and one week of assessments, respectively. ICC calculations use all available data for the one year calculation and subset on the two assessments one week apart for the one week calculation. The subject ID was used as the random effect (i.e. clustering variable) in the mixed model to estimate ICC.

| Autoantibodies | | | T cell subset frequencies | | | CBC | | | NK subset frequencies | | | Antigen Specific T cells (CD4) | | | Antigen Specific T cells (CD8) | | | Monocyte subset frequencies | | | ELISpot/Fluorospot | | |
|---|---|---|---|---|---|---|---|---|---|---|---|---|---|---|---|---|---|---|---|---|---|---|---|
| | 1Year | 1Week | | 1Year | 1Week | | 1Year | 1Week | | 1Year | 1Week | | 1Year | 1Week | | 1Year | 1Week | | 1Year | 1Week | | 1Year | 1Week |
| ZNT8 | 0.99 | 1 | CD8 N | 0.94 | 0.97 | MCV | 0.86 | 0.95 | NK CELLS CD2+ | 0.96 | 0.98 | CD4+AGTMR +CD45RO+ | 0.62 | 0.87 | Sum of AgSpc CD8 TEMRA CXCR3+ | 0.57 | 0.79 | CD14HI MONO HLA CLASS II+ | 0.76 | 0.86 | IL-2 - AR POOL | 0.41 | 0.97 |
| IA2 | 0.96 | 0.99 | CD4 N | 0.94 | 0.97 | MCH | 0.86 | 0.97 | NK CELLS CD57+ | 0.94 | 0.94 | CD4+AGTMR + | 0.61 | 0.86 | Sum of AgSpc CD8 TEMRA | 0.57 | 0.78 | CD14LO MONO PDL1+ | 0.73 | 0.75 | IFNG - QDM | 0.4 | 0 |
| GAD | 0.96 | 0.98 | CD8 TEMRA | 0.93 | 0.97 | PLATELET COUNT | 0.84 | 0.86 | NKHI CELLS CD2+ | 0.9 | 0.94 | CD4+AGTMR +CD45RO+ | 0.55 | 0.58 | Sum of AgSpc CD8 TEMRA CXCR3+ | 0.53 | 0.79 | CD14HI MONO PDL1+ | 0.68 | 0.68 | IFNG - AR POOL | 0.27 | 0.56 |
| IAA | 0.89 | 0.99 | CD4 CXCR3+ | 0.89 | 0.95 | ABS. LYMPHOCYTES | 0.82 | 0.84 | NK CELLS CD54+ | 0.83 | 0.72 | | | | Sum of AgSpc CD8 TEMRA | 0.49 | 0.79 | CD14HI MONO CD2+ | 0.63 | 0.44 | IFNG/IL-2 - AR POOL | 0.22 | 0.89 |
| | | | CD8 EM | 0.88 | 0.94 | HEMOGLOBIN | 0.81 | 0.85 | NKHI CELLS NKG2D+ | 0.78 | 0.81 | | | | Sum of AgSpc CD8 EM | 0.48 | 0.5 | CD14LO MONOCYTES CD2+ | 0.6 | 0.48 | IL-2 - INS POOL | 0.18 | 0.79 |
| | | | CD8 CM | 0.87 | 0.94 | RED BLOOD CELL COUNT | 0.8 | 0.81 | NKHI CELLS PDL1+ | 0.76 | 0.82 | | | | Sum of AgSpc CD8 EM CXCR3+ | 0.44 | 0.67 | CD14HI MONO CD57+ | 0.54 | 0.61 | IL-10- INS POOL | 0.15 | 0.23 |
| | | | CD8 | 0.85 | 0.93 | HEMATOCRIT | 0.78 | 0.8 | NKHI CELLS | 0.73 | 0.81 | | | | Sum of AgSpc CD8 EM | 0.44 | 0.56 | CD14LO MONO CD57+ | 0.52 | 0.76 | IL-10- AR POOL | 0.14 | 0 |
| | | | CD4 CM | 0.85 | 0.93 | PERCENT EOSINOPHILS | 0.73 | 0.82 | NKHI CELLS CD57+ | 0.72 | 0.49 | | | | Sum of AgSpc CD8 EM CXCR3+ | 0.44 | 0.61 | CD14LO MONO HLA CLASS II+ | 0.48 | 0.5 | IL-10- QDM | 0.12 | 0.03 |
| | | | CD4 TEMRA | 0.82 | 0.78 | ABS. EOSINOPHILS | 0.72 | 0.88 | NK CELLS PDL1+ | 0.7 | 0.87 | | | | Sum of AgSpc CD8 CM | 0.35 | 0.67 | CD14HI MONOCYTES | 0.43 | 0.45 | IFNG - INS POOL | 0.12 | 0.02 |
| | | | CD4 EM | 0.82 | 0.91 | WBC COUNT | 0.69 | 0.87 | NKHI CELLS CD54+ | 0.65 | 0.75 | | | | Sum of AgSpc CD8 CM CXCR3+ | 0.33 | 0.56 | CD14LO MONOCYTES | 0.38 | 0.35 | IL-2 - QDM POOL | 0.11 | 0.96 |
| | | | CD8 CXCR3+ | 0.76 | 0.86 | ABS. MONOCYTES | 0.67 | 0.82 | NK CELLS | 0.6 | 0.81 | | | | Sum of AgSpc CD8 CM | 0.3 | 0.56 | CD14LO MONO CD36+ | 0.25 | 0.14 | IFNG/IL-2 - INS POOL | 0.09 | 0.8 |
| | | | CD4 | 0.63 | 0.71 | RDW | 0.65 | 0.91 | NK CELLS CD36+ | 0.47 | 0.68 | | | | Sum of AgSpc CD8 CM CXCR3+ | 0.25 | 0.44 | CD14HI MONO CD36+ | 0.13 | 0.5 | IFNG/IL-2 - QDM | 0.07 | 0 |
| | | | | | | PERCENT MONOCYTES | 0.58 | 0.7 | NK CELLS NKG2D+ | 0.47 | 0.66 | | | | | | | | | | | | |
| | | | | | | PERCENT LYMPHOCYTES | 0.56 | 0.62 | NKHI CELLS NKP46+ | 0.36 | 0.47 | | | | | | | | | | | | |
| | | | | | | ABS. NEUTROPHILS | 0.54 | 0.7 | NKHI CELLS CD36+ | 0.34 | 0.51 | | | | | | | | | | | | |
| | | | | | | PERCENT NEUTROPHILS | 0.53 | 0.62 | NK CELLS NKP46+ | 0.32 | 0.21 | | | | | | | | | | | | |
| | | | | | | PERCENT BASOPHILS | 0.43 | 0.47 | | | | | | | | | | | | | | | |
| | | | | | | ABS. BASOPHILS | 0.4 | 0.52 | | | | | | | | | | | | | | | |
| | | | | | | MCHC | 0.39 | 0.56 | | | | | | | | | | | | | | | |

**1.6 Supplementary Figure S6. Mean and range of ICC values for all subjects compared to T1D, T2D, or controls.** The ICC for all measured markers is displayed by immune marker assay. Dots equal the mean ICC value across all analytes in the assay. Bars indicate the range of values for all analytes measured in the assay. The exception to the broad similarity seen across subject groups is islet autoantibodies, where the difference in ICC is a result of the healthy controls and T2Ds having few/no positive autoantibodies and therefore little variability to explain at the subject level.

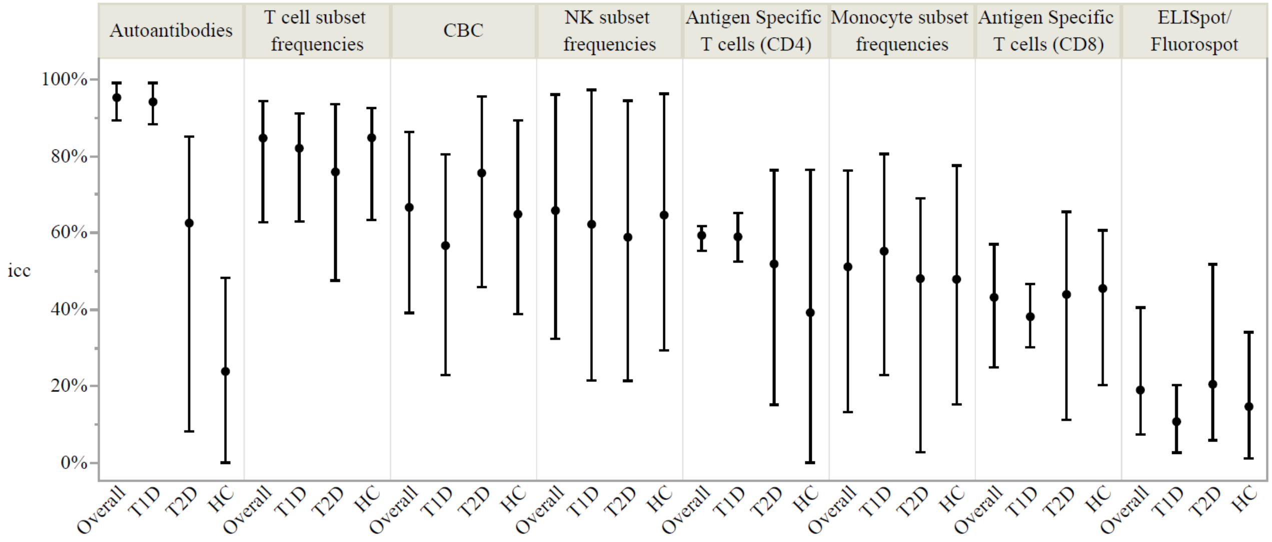

1.7 **Supplementary Figure S7. Little change in insulin secretion over 1 year in T1D cohort.** Individual-level data for insulin secretion as measured by C-peptide in all T1D subjects, where each panel represents one subject and each dot represents the AUC C-peptide from one mixed meal tolerance test (MMTT). X-axis represents visit month at which each MMTT was conducted. Subjects are ordered by insulin secretion at the first MMTT. Subject 25 showed no insulin secretion at first MMTT; the subject continued in the study, but no further MMTTs were conducted.

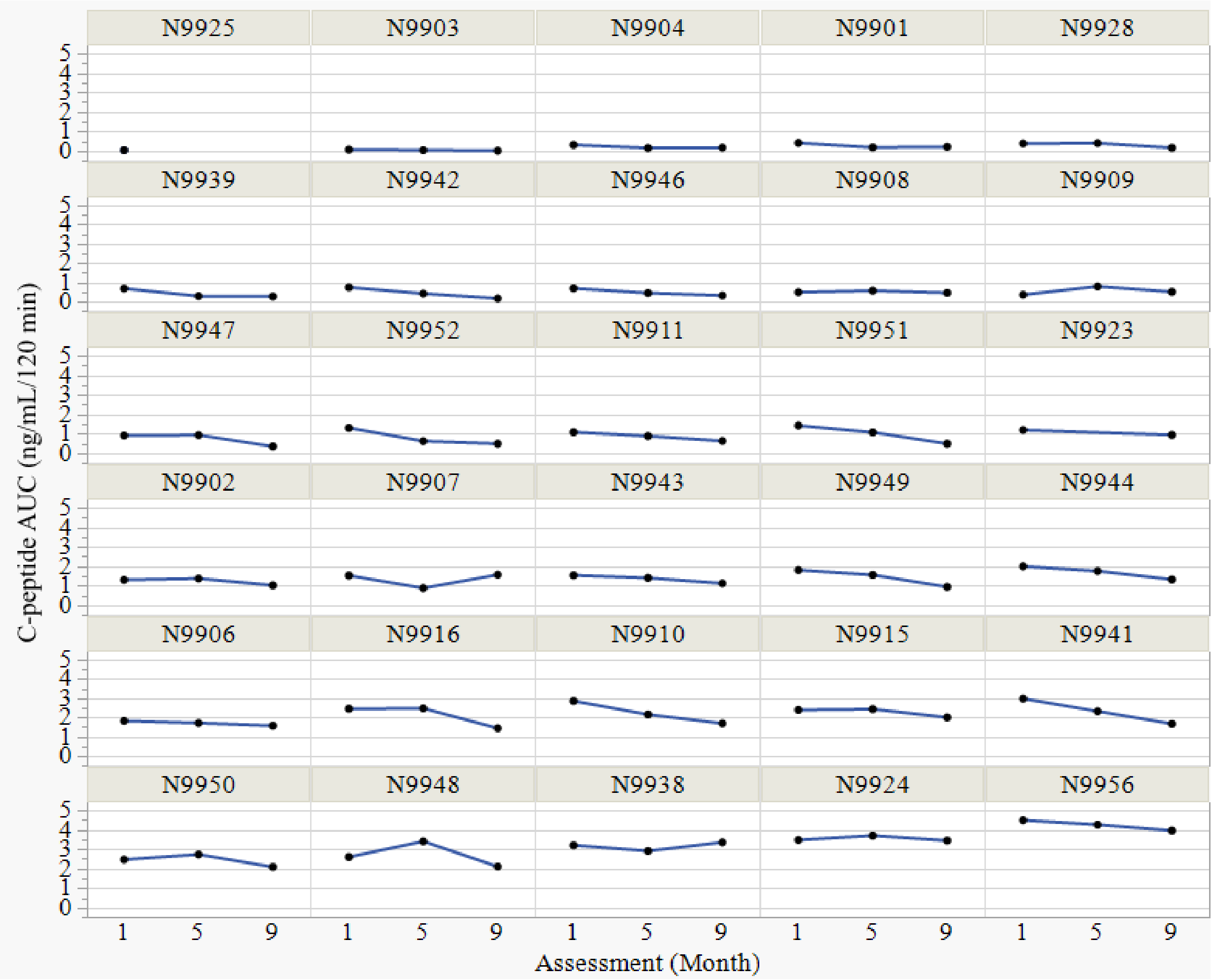

# 2 Supplementary Tables

## 2.1 Supplementary Table S1. Schedule of assessments for longitudinal biomarker study.

| Visit number | 1 | 2 | 3 | 4 | A | 5 | 6 | 7 | 8 | 9 |
|---|---|---|---|---|---|---|---|---|---|---|
| Study months | 0 | 1 | 2 | 3 | 3 | 4 | 5 | 7 | 9 | 11 |
| Subject-related information | | | | | | | | | | |
| Informed consent (IC) | x | | | | | | | | | |
| Inclusion/exclusion criteria | x | | | | | | | | | |
| Clinical, demographic, medical history data | x | | | | | | | | | |
| Height/Weight | x | x | x | x | x | x | x | x | x | x |
| Low- and high-resolution HLA (human leukocyte antigen) genotyping | x | | | | | | | | | |
| Safety | | | | | | | | | | |
| Withdrawal criteria | x | x | x | x | x | x | x | x | x | x |
| Adverse events (AE) | x | x | x | x | x | x | x | x | x | x |
| Concomitant medication | x | x | x | x | x | x | x | x | x | x |
| Complete blood count with differential | x | x | x | x | x | x | x | x | x | x |
| Diabetes data | | | | | | | | | | |
| MMTT Glucose and C-peptide (T1D only) | | x | | | | | x | | x | |
| Glucose and C-peptide (T1D, T2D, controls) | x | x | x | x | x | x | x | x | x | x |
| Time and dose of insulin administration and last meal (T1D) | x | x | x | x | x | x | x | x | x | x |
| HbA1c (T1D, T2D, controls) | x | x | x | x | x | x | x | x | x | x |
| Biomarker measurements | | | | | | | | | | |
| Islet autoantibodies | x | x | x | x | x | x | x | x | x | x |
| Antigen specific T-cells (CD4 and CD8) | x | x | x | x | x | x | x | x | x | x |
| Flow-based phenotyping of PBMC subsets (T, Monocyte, NK) | x | x | x | x | x | x | x | x | x | x |
| Functional T-cell assay (ELIspot, FluoroSpot) | x | x | x | x | x | x | x | x | x | x |

## 2.2 Supplementary Table S2. Multimer-Qdot combinations used for antigen-specific CD8 T-cell assay.

| pMHC Monomer | Peptide Sequence | Qdot Multimer #1 | Qdot Multimer #2 |
|---|---|---|---|
| **CMV pp65** | NLVPMVATV | Qdot 585 | Qdot 800 |
| **EBV LMP2** | CLGGLLTMV | Qdot 585 | Qdot 800 |
| **Measles H250** | SMYRVFEVGV | Qdot 585 | Qdot 800 |
| **IGRP** | VLEGLGEAL | Qdot 585 | Qdot 655 |
| **InsB10-18** | HLVEALYLV | Qdot 605 | Qdot 655 |
| **ppIAPP** | KLQVFLIVL | Qdot 605 | Qdot 800 |
| **PPI** | ALWGPDPAAA | Qdot 655 | Qdot 705 |
| **GAD65** | VMNILLQYVV | Qdot 585 | Qdot 705 |
| **IA2** | MVWESGCTV | Qdot 585 | Qdot 605 |

## 2.3 Supplementary Table S3. Summary of assay characteristics detailing antibody clones, vendors, LSRII laser and filters used for the QDM flow cytometry panel.

| | | | | |
|---|---|---|---|---|
| **Purpose** | Determine the frequency of diabetogenic effector, effector memory, & TEMRA CD8 T-cells | | | |
| **Species** | Human | | | |
| **Cell type(s)** | PBMC (cryopreserved) | | | |
| **References** | Hadrup et al. 2009, Velthuis et al. 2010, Andersen et al. 2012 | | | |
| **Instrument** | LSRII | **HTS or Tube?** | Tube | |
| | | | | |
| **Antibody (Clone)** | **Fluorochrome** | **Vendor** | **Laser** | **Filters** |
| Anti-CD8 (RPA-T8) | APC | BD Pharmigen | Red | 670/30 No LP |
| Anti-CD4 (SKe) | APC-H7 | BD Biosciences | Red | 780/60 750 LP |
| Anti-CD14 (HCD14) | FITC | BD Biosciences | Blue | 525/50 505 LP |
| Anti-CD16 (3G8) | FITC | BD Pharmigen | Blue | 525/50 505 LP |
| Anti-CD20 (2H7) | FITC | BD Biosciences | Blue | 525/50 505 LP |
| Anti-CD40 (5C3) | FITC | BD Pharmigen | Blue | 525/50 505 LP |
| Anti-CD56 (NCAM16.2) | FITC | BD Biosciences | Blue | 525/50 505 LP |
| Anti-CD45RA (H1100) | Alexa Fluor 700 | BD Biosciences | Red | 720/40 685 LP |
| Anti-CD197 (150503) | BV450 | BD Biosciences | Violet | 450/50 No LP |
| Anti-CD183 (IC6) | PE | BD Pharmigen | Yellow/Green | 582/15 555 LP |
| LDA | AmCyan | Life Technologies | Violet | 525/50 505 LP |

## 2.4 Supplementary Table S4. QDM Phenotyping Marker Definitions, Excluding Multimers, and Use.

| Marker | Primary Definition | Use in Panel | Select Ref |
|---|---|---|---|
| **QDM** | | | |
| **CD8** | Co-receptor for the major cytolytic population of T-cells | Subset | Appay et al. 2008. *Cytometry A*. 73(11): 975-983 |
| **CD4** | Co-receptor for a helper T-cells; important for Ab responses & CD8 T-cell activation; includes regulatory cells | Subset | Appay et al. 2008. Cytometry A. 73(11): 975-983 |
| **CD45RA** | Marker of naïve T-cells; may be found on terminally differentiated & transitional memory cells | Maturation, activation, & subset | Arlettaz et al. 1999. *Eur J Imm*. 29(12): 3987-3994; Wills et al. 1999. *J Imm*. 7080-7087; Appay et al. 2008. Cytometry A. 73(11): 975-983. |
| **CD197** | Typically expressed on naïve & CM cells, lost on EM & may be re-expressed on TEMRA; alternative name, CCr7 | Maturation & activation state; function | Sallusto et al. *Nature*. 1999. 401: 708-712; Appay et al. 2008. Cytometry A. 73(11): 975-983 |
| **CD183** | Chemokine receptor expressed by Th1-skewed T-cells or cells that give rise to a subset involved in Th1 inflammation; alternative name CxCr3 | Maturation & activation state; function | Rivino et al. 2004. *J Exp Med*. 20: 200(6): 725-735; Frigerio et al. 2002. *Nat Med*. 8:1414-1420; Appay et al. 2008. Cytometry A. 73(11): 975-983 |
| **CD14** | Expressed on macrophages &, to a lesser degree, neutrophils & DCs | Monocyte exclusion | |
| **CD16** | Expressed by NK cells, neutrophils, monocytes, & macrophages; triggers NK-mediated lysis & is important for ADCC; early marker of NK activation/maturation | Monocyte & NK exclusion | |
| **CD20** | Main B-cell marker | B-cell Exclusion | |
| **CD40** | Expressed by professional, mature APC | APC exclusion | |
| **CD56** | CD56brightCD16- cells potent cytokine producers, low cytolytic but highly proliferative; CD56dimCD16+ are highly cytolytic | NK exclusion | |

## 2.5 Supplementary Table S5. Summary of assay characteristics detailing antibody clones, vendors, LSRII laser and filters used for the NK/monocyte flow cytometry panel.

| | | | | |
|---|---|---|---|---|
| **Purpose** | Phenotype basic monocyte and NK cell subsets ex vivo | | | |
| **Species** | Human PBMC | | | |
| **Cell type(s)** | NK cells | | | |
| **Instrument** | LSR II | **HTS or Tube?** | Either | |
| | | | | |
| **Antibody (Clone)** | **Fluorochrome** | **Vendor** | **Laser** | **Filters** |
| **CD57 (HCD57)** | BV421 (Pacific Blue) | Biolegend | Violet | 450/50 (no LP) |
| **CD36 (CB38)** | BV605 | BD | Violet | 610/20 (595 LP) |
| **CD14 (M5E2)** | BV711 | Biolegend | Violet | 710/40 (685 LP) |
| **HLA-DR,DQ,DP (Tu39)** | FITC | BD | Blue | 525/50 (505 LP) |
| **CD206 (15-2)** | PerCP-Cy5.5 | Biolegend | Blue | 695/40 (635 LP) |
| **NKP46 (9E2/NKP46)** | PE | BD | Yellow-Green | 582/15 (no LP) |
| **CD3 (UCHT1)** | ECD (PE-TxRD) | Beckman coulter | Yellow-Green | 610/20 (600 LP) |
| **CD19 (J3-119)** | ECD (PE-TxRD) | Beckman coulter | Yellow-Green | 610/20 (600 LP) |
| **CD54 (HA58)** | PE-Cy5 | BD | Yellow-Green | 670/30 (635 LP) |
| **CD56 (NCAM16.2)** | PE-Cy7 | BD | Yellow-Green | 780/60 (750 LP) |
| **NKG2D (1D11)** | APC | BD | Red | 670/30 (no LP) |
| **CD16 (3G8)** | APC-H7 (APC-Cy7) | BD | Red | 780/60 (750 LP) |
| **CD2 (RPA-2.10)** | BUV395 | BD | UV | 379/28 (no LP) |
| **CD274 (MIH1)** | BV786 (Qdot 800) | BD | Violet | 800/30 (750 LP) |
| **LDA** | AmCyan | Life Technologies | Violet | 525/50 (505 LP) |

## 2.6 Supplementary Table S6. NK/Monocyte Panel Marker Description and Use.

| Marker | Primary Definition | Use in Panel | Select Ref |
|---|---|---|---|
| **NK/Mono** | | | |
| **CD57** | Marker of senescent & terminal differentiation; mostly explored on NK cells | Maturation & activation state | Lopez-Verges et al. 2010. *Blood.* 116: 3865-3874 |
| **CD36** | Scavenger receptor on monocytes, recognizes oxidized phospholipids and lipoproteins; participates in internalization of apoptotic cells; mainly used to evaluate Monocyte subsets; some evidence NK expression indicates circulating NK precursors | Subset; function | Chen et al. 2015. *Scientific Reports.* 5: 15118 |
| **CD14** | Co-receptor for LPS (along with TLR4 & 2); functions as a pattern recognition receptor; expressed on macrophages &, to a lesser degree, neutrophils & DCs; soluble form may be secreted by liver & monocytes | Main Subset id | Guilliams et al. 2014. *Nat Rev Imm.* 14(8):571-578 |
| **HLA-DR,DQ,DP** | Antigen-presentation, HLA class II (monocyte) | Maturation & activation state; function | Guilliams et al. 2014. *Nat Rev Imm.* 14(8):571-578 |
| **CD206** | C-type lectin mostly expressed on macrophages & immature DC; involved in pathogen phagocytosis & Ag uptake; scavenger receptor important for removing mannoglycoproteins to resolved inflammation | Subset; function | Guilliams et al. 2014. *Nat Rev Imm.* 14(8):571-578 |
| **NKP46** | Activating receptor; maturation marker, correlates with cytolytic capacity (NK cells) | Subset, function, maturation | Freud and Caligiuri, 2006. *Imm Reviews. 214: 56-72*. Michel et al., 2016. J Imm 196: 2923-2931; Poli et al. 2009. Immunology. 126(4): 458-465. |
| **CD54** | Adhesion molecule involved in tissue transmigration; constitutively expressed at low level on monocytes/macs, lymphocytes, granulocytes, DC, & activated endothelial cells, upregulated following activation; ligand for LFA-1; co-stimulator for NK cell-mediated cytotoxicity | Maturation & activation state; function | Wang et al. 2008. *Blood*. 111:1456-1463; Poli et al. 2009. *Immunology.* 126(4): 458-465; Grisar et al. *Arthritis Res*. 3(2):127-132. |
| **CD56** | CD56brightCD16- cells potent cytokine producers, low cytolytic but highly proliferative; CD56dimCD16+ are highly cytolytic | Main Subset id; maturation & function | Lanier et al., 1991. *J Imm* 146: 4421-4426; Michel et al., 2016. *J Imm* 196: 2923-2931 |
| **NKG2D** | Homodimer associated with DAP10; expressed on NK, CD8 T-cells; recognized induced self-proteins to regulate cell activation | Subset; function | Freud and Caligiuri, 2006. *Imm Reviews.* 214: 56-72 |
| **CD16** | Expressed by NK cells, neutrophils, monocytes, & macrophages; triggers NK-mediated lysis & is important for ADCC; early marker of NK activation/maturation | Main Subset id; maturation & function | Freud and Caligiuri, 2006. *Imm Reviews.* 214: 56-72; Chen et al. 2015. Scientific Reports. 5: 15118 |
| **CD2** | Adhesion molecule, expressed by mature activated cells | Subset; function | Freud and Caligiuri, 2006. *Imm Reviews.* 214: 56-72; Poli et al. 2009. Immunology. 126(4): 458-465. |
| **CD274** | PDL1; mediates suppression or indicates functional state | Subset; function | Michel et al., 2016. J Imm 196: 2923-2931; Dong et al. *Cancer Discovery,* in press.; Bianchini et al. 2019. *Science Imm*. 4(36) |
| **CD3** | Main T-cell marker | T-cell Exclusion | |
| **CD19** | Main B-cell marker | B-cell Exclusion | |

## 2.7 Supplementary Table S7. Peptides used for antigen-specific CD4 T-cell assay.

| DR0401 Tetramers | Peptide Sequence | Fluorochrome |
|---|---|---|
| **DR0401/Preproinsulin 88S** | SLQPLALEGSLQSRG | PE |
| **DR0401/GAD65 557I** | NFIRMVISNPAAT | PE |
| **DR0401/GAD65 p15** | DVMNILLQYVVKSFDRSTKV | PE |
| **DR0401/GAD65 p34** | KGMAALPRLIAFTSEHSHFS | PE |

## 2.8 Supplementary Table S8. Peptide pools used for the ELISpot and FluoroSpot assays. Viral pool serves as a positive control for antigen reactivity. Autoreactive (AR) pool composed of peptides from diabetes antigens specific for multiple HLA Class II haplotypes to look at autoreactive CD4 T cells. QDM pool composed of diabetes-associated peptides to assess CD8 T cell responsiveness. JPT = JPT Peptide Technologies.

| Peptide Pool | Peptide | Sequence | Source |
|---|---|---|---|
| Viral | NS1 34-42 | DRLRRDQKS | JPT |
| Viral | TT 1273-1284 | GQIGNDPNRDIL | JPT |
| Viral | NP 206-229 | FWRGENGRKTRIAYERMCILKGK | JPT |
| Viral | TT 830-844 | QYIKANSKFIGITEL | JPT |
| Viral | MP 62-70 | GFVFTLTVPSER | JPT |
| Viral | HA 307-319 | PKWVKQNTLKLAT | JPT |
| Viral | CMV pp65 pool (CMV Peptivator) | 15-mer, 11AA overlap | Miltenyi Biotec |
| Viral | EBV EBNA-1 pool (EBV Peptivator) | 15-mer, 11AA overlap | Miltenyi Biotec |
| AR | InsB 11-23 22E | LVEALYLVCGEEG | JPT |
| AR | PPI 48-60 | FFYTPKTRREAED | JPT |
| AR | PPI 64-76 | GQVELGGGPGAGS | JPT |
| AR | PPI 69-88 | GGGPGAGSLQPLALEGSLQK | JPT |
| AR | PPI 78-90 88S | QPLALEGSLQSRG | JPT |
| AR | GAD 115-127 120E | MNILLEYVVKSFD | JPT |
| AR | GAD 121-140 | YVVKSFDRSTKVIDFHYPNE | JPT |
| AR | GAD 250-266 | AMMIARFKMFPEVKEKG | JPT |
| AR | GAD 265-284 | KGMAALPRLIAFTSEHSHFS | JPT |
| AR | GAD 335-352 | TAGTTVYGAFDPLLAVAD | JPT |
| AR | GAD 555-567 557i | NFIRMVISNPAAT | JPT |
| AR | IGRP 8-27 | GVLIIQHLQKDYRAYYTFLN | JPT |
| AR | IGRP 225-244 | LRVLNIDLLWSVPIAKKWCA | JPT |
| AR | IGRP 241-260 | KWCANPDWIHIDTTPFAGLV | JPT |
| AR | IA2 709-736 | LAKEWQALCAYQAEPNTCATAQGEGNIK | JPT |
| AR | IA2 853-872 | SFYLKNVQTQETRTLTQFHF | JPT |
| QDM | CMV pp65 | NLVPMVATV | JPT |
| QDM | EBV LMP2 | CLGGLLTMV | JPT |
| QDM | Measles H250 | SMYRVFEVGV | JPT |
| QDM | IGRP | VLFGLGFAI | JPT |
| QDM | InsB10-18 | HLVEALYLV | JPT |
| QDM | ppIAPP | KLQVFLIVL | JPT |
| QDM | PPI | ALWGPDPAAA | JPT |
| QDM | GAD65 | VMNILLQYVV | JPT |
| QDM | IA2 | MVWESGCTV | JPT |

## 2.9 Supplementary Table S9. NK/Monocyte Flow Cytometry Panel Population Definitions.

| Population | Definition | Details |
|---|---|---|
| Live Cells | Singlets, Live | Percentages of single live cells |
| NK Cells | Singlets, Live, Dump-, size, CD14-, CD56+, CD16+ | Percentage of CD14- Lymphocyte events |
| NK Cells CD2+ | Singlets, Live, Dump-, size, CD14-, CD56+, CD16+, CD2+ | Percentage of NK cells |
| NK Cells CD36+ | Singlets, Live, Dump-, size, CD14-, CD56+, CD16+, CD36+ | Percentage of NK cells |
| NK Cells CD54+ | Singlets, Live, Dump-, size, CD14-, CD56+, CD16+, CD54+ | Percentage of NK cells |
| NK Cells CD57+ | Singlets, Live, Dump-, size, CD14-, CD56+, CD16+, CD57+ | Percentage of NK cells |
| NK Cells NKG2D+ | Singlets, Live, Dump-, size, CD14-, CD56+, CD16+, NKG2D+ | Percentage of NK cells |
| NK Cells NKp46+ | Singlets, Live, Dump-, size, CD14-, CD56+, CD16+, NKp46+ | Percentage of NK cells |
| NK Cells PDL1+ | Singlets, Live, Dump-, size, CD14-, CD56+, CD16+, PDL1+ | Percentage of NK cells |
| NKhi Cells | Singlets, Live, Dump-, size, CD14-, CD56hi, CD16- | Percentage of CD14- Lymphocyte events |
| NKhi Cells CD2+ | Singlets, Live, Dump-, size, CD14-, CD56hi, CD16-, CD2+ | Percentage of NKhi Cells |
| NKhi Cells CD36+ | Singlets, Live, Dump-, size, CD14-, CD56hi, CD16-, CD36+ | Percentage of NKhi Cells |
| NKhi Cells CD54+ | Singlets, Live, Dump-, size, CD14-, CD56hi, CD16-, CD54+ | Percentage of NKhi Cells |
| NKhi Cells CD57+ | Singlets, Live, Dump-, size, CD14-, CD56hi, CD16-, CD57+ | Percentage of NKhi Cells |
| NKhi Cells NKG2D+ | Singlets, Live, Dump-, size, CD14-, CD56hi, CD16-, NKG2D+ | Percentage of NKhi Cells |
| NKhi Cells NKp46+ | Singlets, Live, Dump-, size, CD14-, CD56hi, CD16-, NKp46+ | Percentage of NKhi Cells |
| NKhi Cells PDL1+ | Singlets, Live, Dump-, size, CD14-, CD56hi, CD16-, PDL1+ | Percentage of NKhi Cells |
| CD14hi Monocytes | Singlets, Live, Dump-, size, CD56-, CD14hi | Percentage of CD56- Monocyte events |
| CD14hi Monocytes CD2+ | Singlets, Live, Dump-, size, CD56-, CD14hi, CD2+ | Percentage of CD14hi Monocyte events |
| CD14hi Monocytes CD36+ | Singlets, Live, Dump-, size, CD56-, CD14hi, CD36+ | Percentage of CD14hi Monocyte events |
| CD14hi Monocytes CD57+ | Singlets, Live, Dump-, size, CD56-, CD14hi, CD57+ | Percentage of CD14hi Monocyte events |
| CD14hi Monocytes HLA CLASS II+ | Singlets, Live, Dump-, size, CD56-, CD14hi, HLA CLASS II+ | Percentage of CD14hi Monocyte events |
| CD14hi Monocytes PDL1+ | Singlets, Live, Dump-, size, CD56-, CD14hi, PDL1+ | Percentage of CD14hi Monocyte events |
| CD14lo Monocytes | Singlets, Live, Dump-, size, CD56-, CD14lo | Percentage of CD56- Monocyte events |
| CD14lo Monocytes CD2+ | Singlets, Live, Dump-, size, CD56-, CD14lo, CD2+ | Percentage of CD14lo Monocyte events |
| CD14lo Monocytes CD36+ | Singlets, Live, Dump-, size, CD56-, CD14lo, CD36+ | Percentage of CD14lo Monocyte events |
| CD14lo Monocytes CD57+ | Singlets, Live, Dump-, size, CD56-, CD14lo, CD57+ | Percentage of CD14lo Monocyte events |
| CD14lo Monocytes HLA CLASS II+ | Singlets, Live, Dump-, size, CD56-, CD14lo, HLA CLASS II+ | Percentage of CD14lo Monocyte events |
| CD14lo Monocytes PDL1+ | Singlets, Live, Dump-, size, CD56-, CD14lo, PDL1+ | Percentage of CD14lo Monocyte events |

## 2.10 Supplementary Table S10. Main T-cell Subsets Flow Cytometry Panel Population Definitions.

| Population | Definition | Details |
|---|---|---|
| Live Cells | Singlet/Live_Dead- | Percentage of single events |
| CD4 | Singlet/Live_Dead-/Dump-/CD4+ | Percentage of Dump- events |
| CD4 CM | Singlet/Live_Dead-/Dump-/CD4+/CD45RA-CCR7+ | Percentage of Total CD4+ T-cells |
| CD4 CXCR3+ | Singlet/Live_Dead-/Dump-/CD4+/CXCR3+ | Percentage of Total CD4+ T-cells |
| CD4 EM | Singlet/Live_Dead-/Dump-/CD4+/CD45RA-/CCR7- | Percentage of Total CD4+ T-cells |
| CD4 N | Singlet/Live_Dead-/Dump-/CD4+/CD45RA+/CCR7+ | Percentage of Total CD4+ T-cells |
| CD4 TEMRA | Singlet/Live_Dead-/Dump-/CD4+/CD45RA+/CCR7- | Percentage of Total CD4+ T-cells |
| CD8 | Singlet/Live_Dead-/Dump-/CD8+ | Percentage of Dump- events |
| CD8 CM | Singlet/Live_Dead-/Dump-/CD8+/CD45RA-CCR7+ | Percentage of Total CD8+ T-cells |
| CD8 CXCR3+ | Singlet/Live_Dead-/Dump-/CD8+/CXCR3+ | Percentage of Total CD8+ T-cells |
| CD8 EM | Singlet/Live_Dead-/Dump-/CD8+/CD45RA-/CCR7- | Percentage of Total CD8+ T-cells |
| CD8 N | Singlet/Live_Dead-/Dump-/CD8+/CD45RA+/CCR7+ | Percentage of Total CD8+ T-cells |
| CD4 TEMRA | Singlet/Live_Dead-/Dump-/CD8+/CD45RA+/CCR7- | Percentage of Total CD8+ T-cells |

## 2.11 Supplementary Table S11. QDM Flow Cytometry Panel Population Definitions, Multimer+ Subsets.

| Population | Definition | Details |
|---|---|---|
| Total CD8 IA2+ | Singlet/Live_Dead-/Dump-/CD8+/CD8 N-/Qdot655-/ Qdot705-/Qdot800-/Qdot585+/ Qdot605+/ | Percentage of Total CD8+ T-cells |
| Total CD8 IA2+ CM | Singlet/Live_Dead-/Dump-/CD8+/CD8 N-/Qdot655-/ Qdot705-/Qdot800-/Qdot585+/ Qdot605+/CD45RA-/CCR7+ | Percentage of Total CD8+ T-cells |
| Total CD8 IA2+ CM CXCR3+ | Singlet/Live_Dead-/Dump-/CD8+/CD8 N-/Qdot655-/ Qdot705-/Qdot800-/Qdot585+/ Qdot605+/CD45RA-/CCR7+/CXCR3+ | Percentage of Total CD8+ T-cells |
| Total CD8 IA2+ EM | Singlet/Live_Dead-/Dump-/CD8+/CD8 N-/Qdot655-/ Qdot705-/Qdot800-/Qdot585+/ Qdot605+/CD45RA-/CCR7- | Percentage of Total CD8+ T-cells |
| Total CD8 IA2+ EM CXCR3+ | Singlet/Live_Dead-/Dump-/CD8+/CD8 N-/Qdot655-/ Qdot705-/Qdot800-/Qdot585+/ Qdot605+/CD45RA-/CCR7-/CXCR3+ | Percentage of Total CD8+ T-cells |
| Total CD8 IA2+ TEMRA | Singlet/Live_Dead-/Dump-/CD8+/CD8 N-/Qdot655-/ Qdot705-/Qdot800-/Qdot585+/ Qdot605+/CD45RA+/CCR7- | Percentage of Total CD8+ T-cells |
| Total CD8 IA2+ TEMRA CXCR3+ | Singlet/Live_Dead-/Dump-/CD8+/CD8 N-/Qdot655-/ Qdot705-/Qdot800-/Qdot585+/ Qdot605+/CD45RA+/CCR7-/CXCR3+ | Percentage of Total CD8+ T-cells |
| Total CD8 PPI+ | Singlet/Live_Dead-/Dump-/CD8+/CD8 N-/ Qdot585-/ Qdot605-/Qdot800-/Qdot655+/ Qdot705+/ | Percentage of Total CD8+ T-cells |
| Total CD8 PPI+ CM | Singlet/Live_Dead-/Dump-/CD8+/CD8 N-/ Qdot585-/ Qdot605-/Qdot800-/Qdot655+/ Qdot705+/CD45RA-/CCR7+ | Percentage of Total CD8+ T-cells |
| Total CD8 PPI+ CM CXCR3+ | Singlet/Live_Dead-/Dump-/CD8+/CD8 N-/ Qdot585-/ Qdot605-/Qdot800-/Qdot655+/ Qdot705+/CD45RA-/CCR7+/CXCR3+ | Percentage of Total CD8+ T-cells |
| Total CD8 PPI+ EM | Singlet/Live_Dead-/Dump-/CD8+/CD8 N-/ Qdot585-/ Qdot605-/Qdot800-/Qdot655+/ Qdot705+/CD45RA-/CCR7- | Percentage of Total CD8+ T-cells |
| Total CD8 PPI+ EM CXCR3+ | Singlet/Live_Dead-/Dump-/CD8+/CD8 N-/ Qdot585-/ Qdot605-/Qdot800-/Qdot655+/ Qdot705+/CD45RA-/CCR7-/CXCR3+ | Percentage of Total CD8+ T-cells |
| Total CD8 PPI+ TEMRA | Singlet/Live_Dead-/Dump-/CD8+/CD8 N-/ Qdot585-/ Qdot605-/Qdot800-/Qdot655+/Qdot705+/CD45RA+/CCR7- | Percentage of Total CD8+ T-cells |
| Total CD8 PPI+ TEMRA CXCR3+ | Singlet/Live_Dead-/Dump-/CD8+/CD8 N-/ Qdot585-/ Qdot605-/Qdot800-/Qdot655+/Qdot705+/CD45RA+/CCR7-/CXCR3+ | Percentage of Total CD8+ T-cells |
| Total CD8 ppiAPP+ | Singlet/Live_Dead-/Dump-/CD8+/CD8 N-/ Qdot585-/ Qdot655-/Qdot705-/ Qdot605+/ Qdot800+/ | Percentage of Total CD8+ T-cells |
| Total CD8 ppiAPP+ CM | Singlet/Live_Dead-/Dump-/CD8+/CD8 N-/ Qdot585-/ Qdot655-/Qdot705-/Qdot605+/ Qdot800+/CD45RA-/CCR7+ | Percentage of Total CD8+ T-cells |
| Total CD8 ppiAPP+ CM CXCR3+ | Singlet/Live_Dead-/Dump-/CD8+/CD8 N-/ Qdot585-/ Qdot655-/Qdot705-/Qdot605+/ Qdot800+/CD45RA-/CCR7+/CXCR3+ | Percentage of Total CD8+ T-cells |
| Total CD8 ppiAPP+ EM | Singlet/Live_Dead-/Dump-/CD8+/CD8 N-/ Qdot585-/ Qdot655-/Qdot705-/Qdot605+/ Qdot800+/CD45RA-/CCR7- | Percentage of Total CD8+ T-cells |
| Total CD8 ppiAPP+ EM CXCR3+ | Singlet/Live_Dead-/Dump-/CD8+/CD8 N-/ Qdot585-/ Qdot655-/Qdot705-/Qdot605+/ Qdot800+/CD45RA-/CCR7-/CXCR3+ | Percentage of Total CD8+ T-cells |
| Total CD8 ppiAPP+ EM TEMRA | Singlet/Live_Dead-/Dump-/CD8+/CD8 N-/ Qdot585-/ Qdot655-/Qdot705-/Qdot605+/ Qdot800+/CD45RA+/CCR7- | Percentage of Total CD8+ T-cells |
| Total CD8 ppiAPP+ EM TEMRA CXCR3+ | Singlet/Live_Dead-/Dump-/CD8+/CD8 N-/ Qdot585-/ Qdot655-/Qdot705-/Qdot605+/ Qdot800+/CD45RA+/CCR7-/CXCR3+ | Percentage of Total CD8+ T-cells |
| Total CD8 GAD65+ | Singlet/Live_Dead-/Dump-/CD8+/CD8 N-/Qdot605-/Qdot655-/Qdot800-/Qdot585+/Qdot705+ | Percentage of Total CD8+ T-cells |
| Total CD8 GAD65+ CM | Singlet/Live_Dead-/Dump-/CD8+/CD8 N-/Qdot605-/Qdot655-/Qdot800-/Qdot585+/Qdot705+/CD45RA-/CCR7+/ | Percentage of Total CD8+ T-cells |
| Total CD8 GAD65+ CM CXCR3+ | Singlet/Live_Dead-/Dump-/CD8+/CD8 N-/Qdot605-/Qdot655-/Qdot800-/Qdot585+/Qdot705+/CD45RA-/CCR7+/CXCR3+ | Percentage of Total CD8+ T-cells |
| Total CD8 GAD65+ EM | Singlet/Live_Dead-/Dump-/CD8+/CD8 N-/Qdot605-/Qdot655-/Qdot800-/Qdot585+/Qdot705+/CD45RA-/CCR7- | Percentage of Total CD8+ T-cells |
| Total CD8 GAD65+ EM CXCR3+ | Singlet/Live_Dead-/Dump-/CD8+/CD8 N-/Qdot605-/Qdot655-/Qdot800-/Qdot585+/Qdot705+/CD45RA-/CCR7-/CXCR3+ | Percentage of Total CD8+ T-cells |
| Total CD8 GAD65+ TEMRA | Singlet/Live_Dead-/Dump-/CD8+/CD8 N-/Qdot605-/Qdot655-/Qdot800-/Qdot585+/Qdot705+/CD45RA+/CCR7- | Percentage of Total CD8+ T-cells |
| Total CD8 GAD65+ TEMRA CXCR3+ | Singlet/Live_Dead-/Dump-/CD8+/CD8 N-/Qdot605-/Qdot655-/Qdot800-/Qdot585+/Qdot705+/CD45RA+/CCR7-/CXCR3+ | Percentage of Total CD8+ T-cells |

## 3 Supplementary Data

### 3.1 Data file S1.

**Data file S1.** Technical replicate control plots of all 64 biomarkers (Data File S1).

## 4 Supplementary Detailed Materials and Methods

### 4.1 Whole blood collection and cryopreservation

At each visit, up to 40 mL whole blood was drawn in heparinized tubes for PBMC isolation; 8.5 mL for serum isolation. Serum was preserved per standard protocols and stored at -80 °C until analyzed. All samples were processed and cryopreserved within 24 hours of collection. Prior to PBMC isolation, 50-mL Leucosep tubes (Griener) were prepared with 15 mL Ficoll-Paque Plus (GE Healthcare) up to 6 months before use per manufacturer's instructions, and stored in the dark at room temperature until use. Blood samples from a single individual were pooled in a 50-mL sterile tube; blood collection tubes were rinsed with PBS and the PBS rinse was added to the pooled blood. The blood sample was further diluted with PBS to a ratio of 1:1 (PBS to Blood) and mixed gently; up to 30 mL diluted blood was pipetted into the prepared Leucosep tube. Tubes were centrifuged at 1000 × *g* for 12 minutes at room temperature with no brake. The platelet/plasma-enriched layer was removed to approximately 5 mm above the cell layer. The cell layer was removed and transferred to a clean, sterile 50-mL conical tube. PBS was added to bring the volume to 50 mL and the sample was centrifuged for 10 minutes at 250 × *g* with a low brake and at room temperature. Supernatant was decanted and the cell pellet was gently resuspended by raking across a tube rack and pipetting in phenol red-free RPMI 1640 media (GIBCO) supplemented with 10 mL 1M HEPES (GIBCO) for counting. After counting, PBMC were cryopreserved in 80% human AB serum (Life Technologies), 20% DMSO (Sigma) at a minimum of 10 x $10^6$ cells per vial per mL. Cryovials were transferred to a cryopreservation container; glycerol-containing filler tubes were added to any empty wells in the cryocontainer to ensure even freezing. Cryocontainers were transferred immediately to -80 °C. After

a minimum of 24 hours or up to 5 days at -80 °C, vials were transferred to $LN_2$ storage and remained in storage until analyses at end of study. All samples were stored in a restricted-access $LN_2$ freezer until evaluated and, during the trial analyses, the different aliquots from the same donor were used for all assay runs.

### 4.2 Thawing cryopreserved PBMC

Cryopreserved PBMCs were rapidly thawed at 37 °C in a water bath, sprayed with 70% ethanol to sterilize, and transferred to a biosafety cabinet. PBMCs were resuspended in 9 mL warm complete media (phenol red-free 1640 RPMI (Gibco)) supplemented with 2% Glutamax (Life Technologies), 2% HEPES (Gibco, Life Technologies) and 10% human AB serum (Life Technologies) containing 15 U Benzonase (Novagen) per mL cell suspension in a 15-mL conical tube (Falcon). PBMC samples were centrifuged for 10 minutes at 300 × *g*. The supernatant was decanted into a bucket containing 10% bleach. For flow cytometry assays, the PBMCs were resuspended in 1 mL of warm complete medium and immediately evaluated for cell concentration and viability using the Guava ViaCount Assay (Millipore). Cells were resuspended at 10 x $10^6$ cells/mL and 100 μL (NK/Monocyte panel) or 200 μL (multimer panel) was transferred to each well of a 96-well round bottom plate.

### 4.3 Qdot-Multimer flow cytometry (QDM)

Antigen-specific CD8+ T-cells were evaluated using a modified version of a previously described method (REF *11*). Peptide-HLA-A02:01 complexes were generated as described by Hadrup et al. (REF *35*). All peptide-HLA monomers were stored at -80 °C until multimerized. Prior to multimerization with quantum dots (Qdots, Life Technologies), the concentration of each monomer was confirmed using a NanoDrop Spectrophotometer (Thermo Scientific). All streptavidin-conjugated quantum dots (Qdots, Life Technologies) were stored at 4 °C and were centrifuged at

13,000 rpm (15871 × *g*) for 1 minute at room temperature with high brake to pellet aggregates prior to use.

Monomers and a 1x aliquot of protease inhibitor cocktail set I (Merck) were thawed on ice. Peptide-HLA-A02:01 complexes were multimerized and labeled with Qdots in a 96-well plate and kept at 4 °C throughout the procedure using a 96-well cold block (Biocision). After thawing, 1 μL protease inhibitor cocktail set I (Merck) was added to 10-μg monomers. Streptavidin-conjugated Qdot reagents (Life Technologies) were diluted 1 to 5 and added to the monomer/protease inhibitor solution and placed on a plate rocker at 4 °C for 20 minutes. This procedure was repeated four more times for a total of five 20-minute incubations of the step-wise addition of streptavidin-conjugated Qdots before D-biotin (Avidity) was added at a final concentration of 80 μM and incubated with agitation at 4 °C for 20 minutes. All streptavidin-conjugated Qdot multimers were pooled into single-use aliquots and stored at -80 °C for up to 6 months. Prior to use, Qdot aliquots were thawed at room temperature and centrifuged at 13,000 rpm (15871 × *g*) for 1 minute with high brake to pellet aggregates. pHLA and Qdot combinations are shown in Supplementary Table 2.

After PBMC were thawed and counted, the cell suspension volume was adjusted to 10 x $10^6$ cells/mL phenol red-free 1640 RPMI (Gibco) supplemented with 2% Glutamax (Life Technologies), 2% HEPES (Gibco, Life Technologies), and 10% human AB serum (Life Technologies). Then, 200 μL cell suspension was added per well (2 x $10^6$ cells/well) to a sterile 96-well round-bottom plate. The plate was centrifuged for 2 minutes at 300 × *g* with high brake at room temperature. The media was removed by quickly decanting the plate and cells were resuspended in either Live/Dead Aqua (Molecular Probes) or PBS (GIBCO) as a control, and incubated in the dark for 30 minutes at room temperature. PBS was added to the wells and the plate was centrifuged twice. After the second centrifugation, cells were incubated with 500-nM dasatinib (LC Laboratories) for 30 minutes at 37

°C. The cells were centrifuged for 2 minutes at 300 × *g* with high brake at room temperature. Multimers were added to the wells and incubated with the cells for 10 minutes at 37 °C; fluorescence minus one (FMO) controls for all multimers were generated by incubation with FACS buffer for 10 minutes at 37 °C instead of multimers. After the 10-minute incubation, cells were incubated in PBS supplemented with 2% human AB serum (Life Technologies) and additional fluorochrome-conjugated antibodies for 30 minute at room temperature; all antibodies and cytometer filter set-up are listed in Supplementary Table 3. Data were acquired on an LSRII (BD Biosciences) and analyzed using FlowJo software (Tree Star, Inc., Ashland, OR). For analyses, dead cells were excluded using the Live/Dead stain. Doublet discrimination was accomplished using forward scatter height versus area dot plots, and monocyte, NK-cell, B-cell, and dendritic cell populations were excluded from the analyses using FITC-conjugated anti-CD14, anti-CD16, anti-CD56, anti-CD20, and anti-CD40. Fluorescence-minus-one (FMO) controls were used to set all gates during analyses. For each sample, $1 \times 10^6$ events were acquired for analyses; for each FMO, $5 \times 10^5$ events were acquired. Four PBMC control samples were stained and collected; two were collected at the start of each plate run and two were collected at the end. Each pair comprised one sample spiked with 0.1% primary CMV-specific and PPI-specific CD8+ T-cell clones (kindly provided by M. Peakman, King's College) and one unspiked, non-diabetic PBMC sample. At the time of this study, other reliable clones could not be obtained for this work. Work prior to this study evaluated the limit of detection for the assay using healthy donor samples spiked with decreasing numbers of primary T-cell clones (2% to 0.0001%), and this was used to determine the assay lower limit of detection on the instruments used for this study (.01%). Data was monitored for assay performance and signal loss over time. The gating schema and representative flow cytometry data are shown in Supplementary Figures 1-2.

### 4.4 LSRII QC Procedure

All experiments for this study were performed on two five-laser 20-parameter LSRII flow cytometers with matching configurations. CST beads were run daily on both machines. Additionally, Ultra Rainbow Fluorescent Particles-Mid Range (Spherotech, Lake Forest, IL) were used daily to QC individual parameters. MFI target values for these beads were previously determined for each parameter. Application-specific optimization of PMT voltages was performed to ensure electronic noise minimally contributed to detected signal as recommended by the manufacturer. PMT voltages were adjusted daily to match predetermined MFI target values.

### 4.5 ELISpot and FluoroSpot assays for IL-10, IFN-γ, and IL-2 detection

Pre-coated human IL-10 ELISpot kits and dual-color IFN-γ and IL-2 FluoroSpot kits were purchased from Mabtech AB (3430-4HPW-10 and FSP-0102-10, respectively; Stockholm, Sweden). Plates were washed five times with 100 µL sterile PBS per well and, then, 200 µL 10% RPMI (phenol red-free 1640 RPMI (Gibco) supplemented with 2% Glutamax (Life Technologies), 2% HEPES (Gibco, Life Technologies), and 10% human AB serum (Sigma)) was added to each well and the plate was incubated for 2 hours at 37 °C to block non-specific binding. After thawing of PBMC samples, cells were resuspended at a concentration of 2 x $10^6$/mL in 10% HS-RPMI and rested for 2 hours at 37 °C. Then, cells were centrifuged at 300 x *g* for 10 minutes and resuspended at a concentration of 2.5 x $10^6$ cells/mL 10% HS-RPMI for ELISpot and 2.0 x $10^6$ cells/mL for FluoroSpot assays. Prior to addition of cells, 0.5 µg PHA/mL media and 0.01% cell culture-grade DMSO were used as positive and negative stimuli, respectively; the peptide pools used are described in Supplementary Table 6. Cells were added to each well (100 µL per well) in triplicate and plates were incubated at 37 °C in 5% $CO_2$ with humidity for 44 - 48 hours. Then, plates were washed five times using the Biotek plate washer prior to addition of the detection Ab per the manufacturer's instructions (IL-10 ELISpot: 12-G8-biotin; IFN-γ/IL-2 FluoroSpot: 7-B6-1-FS-FITC and MT8G10-biotin). Plates were incubated for

2 hours at room temperature in the dark and washed five times with Biotek plate washer to remove the detection Ab. For the ELISpot, 100 µL streptavidin-HRP (1:1000) in 0.1% BSA-PBS was added to each well and the plate was incubated for 1 hour at room temperature prior to washing and adding 100 µl TMB substrate per well for spot development. For the FluoroSpot, 100 µL of the secondary detection was added to each well (anti-FITC-490 & SA-550 (1:200)) and plates were incubated for 1 hour at room temperature. After washing, 50 µL fluorescent enhancer solution was added to each well and the plate was incubated for another 15 minutes at room temperature. The plastic basin was removed from the underside of the membrane for both ELISpot and FluoroSpot plates and all plates were rinsed with water to stop development or removed enhancer solution. All plates were allowed to air-dry fully (at least 60 hours post-assay completion) in the dark before being analyzed using the AID multispot reader.

The number of spot-forming cells (SFCs) in the ELISpot and FluoroSpot assays were analyzed and counted using the FluoroSpot reader system (AID ELISpot Reader version 7.0 build 14790, AID GmbH, Strassberg Germany). Camera exposure and gain were modified for each filter to obtain the best exposure of spot images. Fluorophore-specific spot parameters including spot size, spot intensity spot gradient and a spot separation algorithm were tuned in the software for optimal spot detection by comparing positive control wells containing PHA-L with negative control wells (media + 0.01% DMSO). Three replicates of each sample were run and averaged to determine the average SFC count per sample that was then normalized by the number of viable cells plated per well (counted using a Guava Easy-Cyte flow cytometer) to determine the average spot count per million.

### 4.6 Code for statistical analyses

The following SAS code was used to calculate ICC values where the ‘tall’ dataset was structured as one record per subject, per repeated measure, per biomarker.

```
proc sort data=novo_long1; by lparm_with_units pt; run;

ods output covparms=cov;

proc mixed data=novo_long1 method=reml covtest;

   where numeric_result ne . ;
     by lparm_with_units;
    class pt  ;
      model numeric_result = ;
       random pt  ;
run;

proc sql;
create table icc1 as
select lparm_with_units, sum(estimate*(covparm='PT'))
/ sum(estimate) as icc
from cov
group by lparm_with_units;
quit ;
```

The following JSL code was used to develop the partial least squares model.

```
Fit Model(

     Y( :Name( "AUC C PEPTIDE (ng/mL/120 min )" ) ),
     Effects(
          :Name( "ZNT8 RESULT (units/mL (NIDDK) )" ),
          :Name( "IA2 RESULT (units/mL (NIDDK) )" ),
          :Name( "IAA RESULT (units/mL (NIDDK) )" ),
          :Name( "CD8 TEMRA (% of CCR7-CD8 cells )" ),
          :Name( "CD4 CXCR3+ (% of CD4 cells )" ),
          :Name( "CD8 EM (% of CCR7-CD8 cells )" ),
          :Name( "CD8 CM (% of CCR7-CD8 cells )" ),
          :Name( "CD8 (% of all cells )" ),
          :Name( "CD8 CXCR3+ (% of CCR7-CD8 cells )" ),
          :Name( "NKHI CELLS CD2+ (% of NKhi cells )" ),
          :Name( "NKHI CELLS NKG2D+ (% of NKhi cells )" ),
          :Name( "NKHI CELLS (% of NK cells )" ),
          :Name( "CD14HI MONO HLA CLASS II+ (% of CD14hi )" ),
          :Name( "CD14LO MONO PDL1+ (MFI of PDL1 on CD14lo )" )
     ),
```

```
    No Intercept( 1 ),
    Center Polynomials( 0 ),
    Personality( "Partial Least Squares" ),
    Run(
        Initial Number of Factors( 14 ),
        Validation Method( KFold( 7 ), Initial Number of Factors(
14 ) ),
        Fit(
            Method( NIPALS ),
            Number of Factors( 1 ),
            Show Confidence Band( 1 ),
            Variable Importance Plot( 1 ),
            Diagnostics Plots( 1 ),
            Overlay Loadings Plots( 1 ),
            VIP vs Coefficients Plots( 1 ),
            Percent Variation Plots( 1 ),
            Overlay Coefficients Plots( 1 )
        )
    ),
    SendToReport(
        Dispatch(
            {"NIPALS Fit with 1 Factors"},
            "Model Coefficients for Centered and Scaled Data",
            OutlineBox,
            {Close( 0 )}
        ),
        Dispatch(
            {"NIPALS Fit with 1 Factors"},
            "Model Coefficients for Original Data",
            OutlineBox,
            {Close( 0 )}
        ),
        Dispatch(
            {"NIPALS Fit with 1 Factors", "Variable Importance
Plot"},
            "Variable Importance Plots",
            FrameBox,
            {Frame Size( 684, 300 )}
        ),
        Dispatch(
            {"NIPALS Fit with 1 Factors", "Variable Importance
Plot",
            "Variable Importance Table"},
            "",
            TableBox,
            {Sort By Column( 2, 0 )}
        ),
        Dispatch(
```

```
			{"NIPALS Fit with 1 Factors", "Diagnostics Plots",
			"Actual by Predicted Plot"},
			"1",
			ScaleBox,
			{Format( "Fixed Dec", 12, 0 ), Min( -0.5 ), Max(
4.1575 ), Inc( 1 ),
			Minor Ticks( 1 )}
		),
		Dispatch(
			{"NIPALS Fit with 1 Factors", "Diagnostics Plots",
			"Actual by Predicted Plot"},
			"2",
			ScaleBox,
			{Min( -0.56625 ), Max( 4.5 ), Inc( 1 ), Minor Ticks(
1 )}
		),
		Dispatch(
			{"NIPALS Fit with 1 Factors", "Diagnostics Plots",
			"Actual by Predicted Plot"},
			"Diagnostics Plots",
			FrameBox,
			{Frame Size( 315, 267 ), Marker Size( 5 )}
		),
		Dispatch(
			{"NIPALS Fit with 1 Factors", "Diagnostics Plots",
			"Residual by Predicted Plot"},
			"1",
			ScaleBox,
			{Format( "Fixed Dec", 12, 0 ), Min( -0.5 ), Max(
4.1575 ), Inc( 1 ),
			Minor Ticks( 1 )}
		),
		Dispatch(
			{"NIPALS Fit with 1 Factors"},
			"Y Loading Plot",
			OutlineBox,
			{Close( 1 )}
		),
		Dispatch(
			{"NIPALS Fit with 1 Factors",
			"VIP vs Coefficients for Centered and Scaled Data"},
			"VIP vs Coefficients",
			FrameBox,
			{Frame Size( 730, 452 )}
		),
		Dispatch(
			{"NIPALS Fit with 1 Factors", "VIP vs Coefficients
for Original Data"},
```

```
                    "VIP vs Coefficients",
                    FrameBox,
                    {Frame Size( 719, 390 )}
                ),
                Dispatch(
                    {"NIPALS Fit with 1 Factors",
                    "Percent Variation Explained for X Effects"},
                    "Percent Variation Explained for X Effects",
                    FrameBox,
                    {Frame Size( 369, 372 )}
                )
            )
        );
```
