# Supplementary material for: Systematic Assessment of Immune Marker Variation in Type 1 Diabetes: A Prospective Longitudinal Study
Source: Front Immunol. 2019 Sep 13;10:2023. doi: 10.3389/fimmu.2019.02023 (PMC6753618; doi:10.3389/fimmu.2019.02023)

Each chart displays the frequency of a given population detected in two replicate aliquots from a single blood draw for each subject (top portion) measured on multiple experiment days (x-axis =study day). Replicate tests were run at the beginning and end of each day. The bottom portion of each control chart displays the range of the two replicate tests for each day. Subject IDs (PTID) are labeled with letters across the top and bottom; multiple and sometimes different control subjects were tested for each immune marker. Green lines represent the mean cell frequency for the two replicate measurements (top portion) and range (bottom portion) for each subject. The red lines represent the upper and lower control limits calculated using the range as the variability estimate. The statistical control limits are calculated per-subject and represent three times the variability estimate divided by the square root of the sample size.

CD4

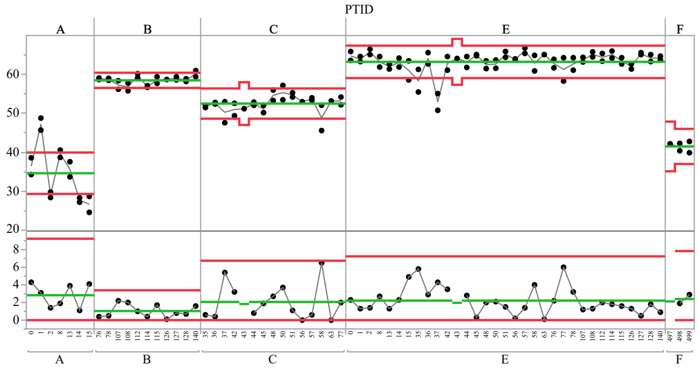

CD8

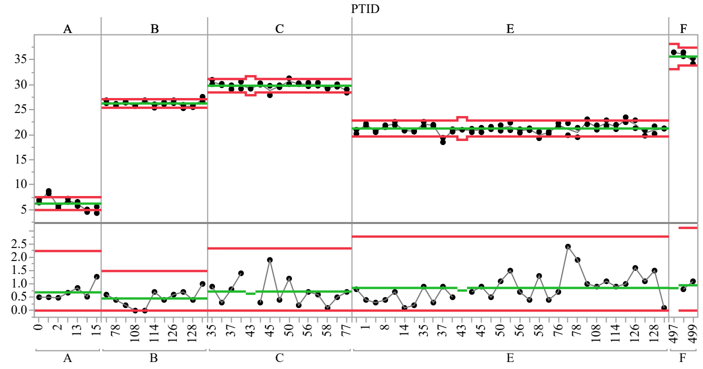

CD14hi Monocytes

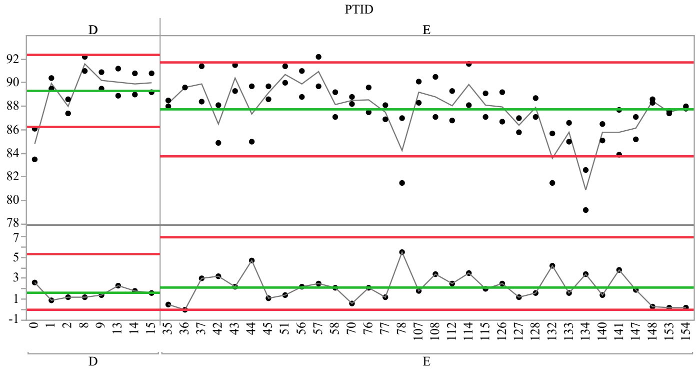

CD14hi Monocytes CD2+

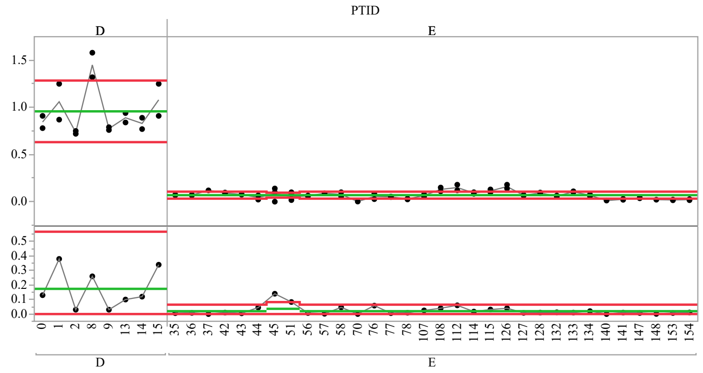

CD14hi Monocytes CD36+

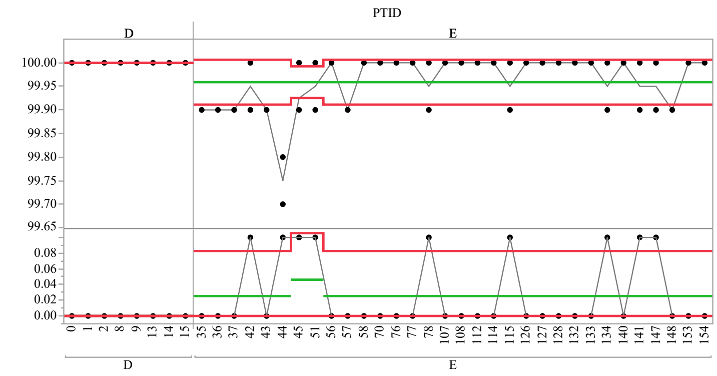

CD14hi Monocytes CD57+

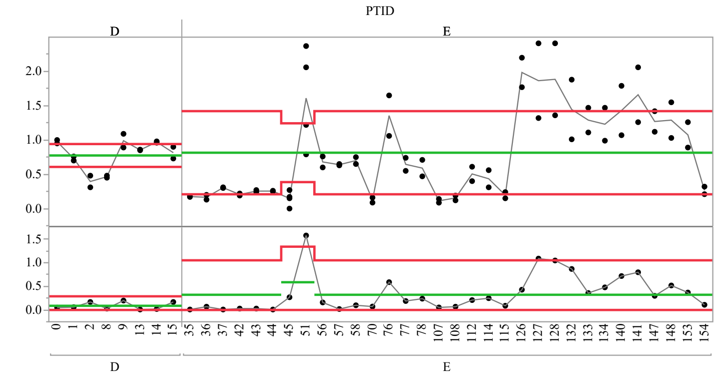

CD14hi Monocytes HLA CLASS II+

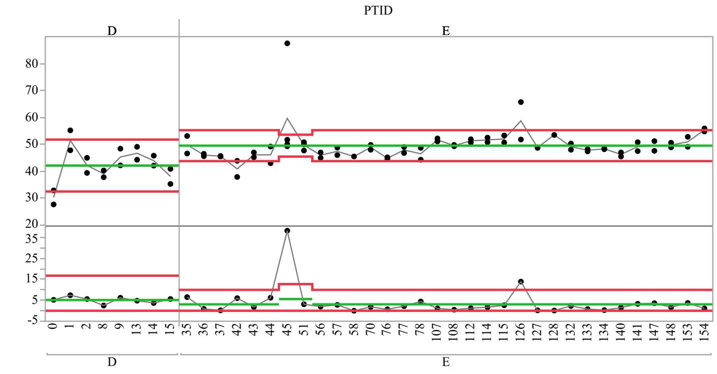

CD14hi Monocytes PDL1 Median

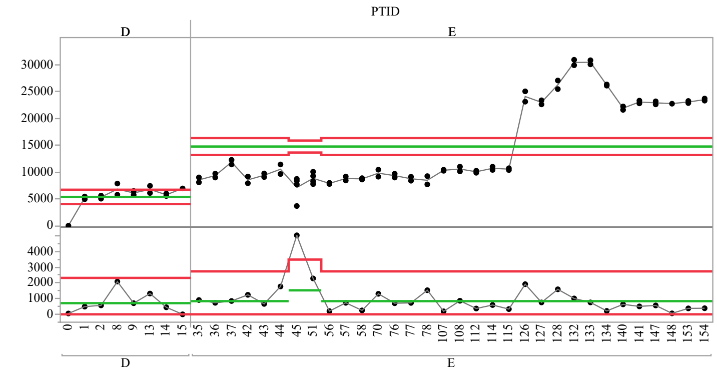

CD14lo Monocytes

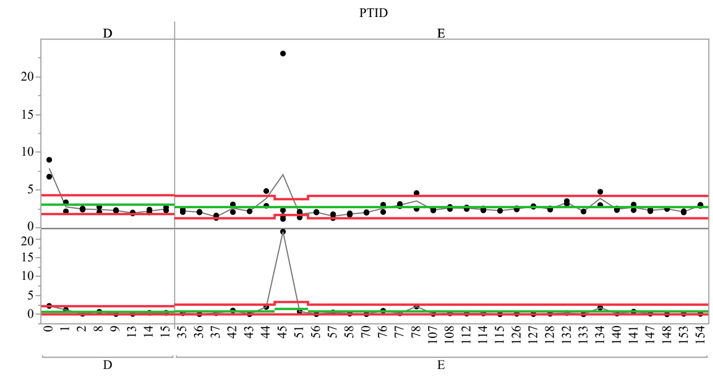

CD14lo Monocytes CD2+

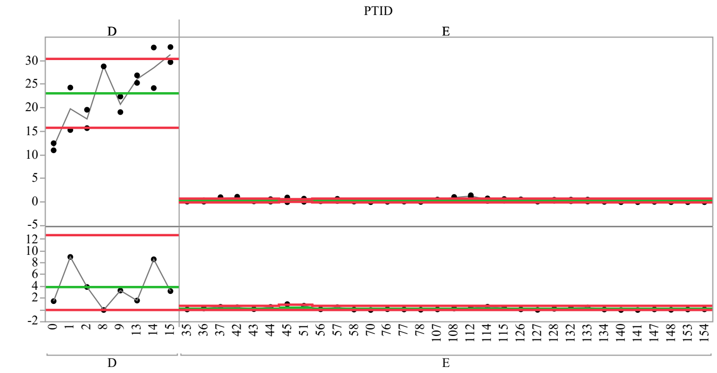

CD14lo Monocytes CD36+

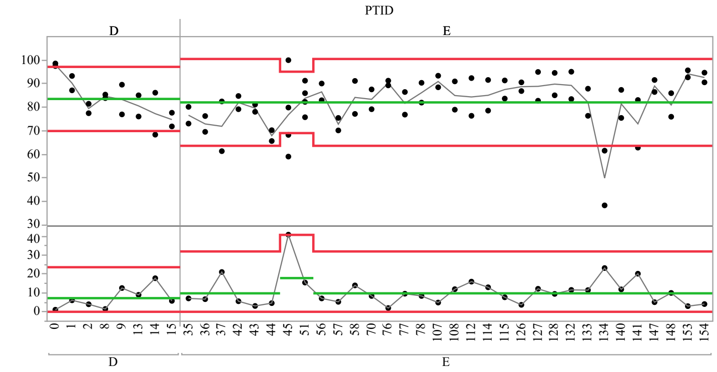

CD14lo Monocytes CD57+

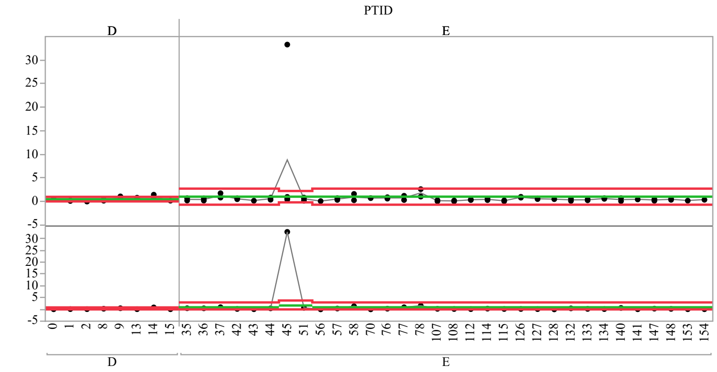

CD14lo Monocytes HLA CLASS II+

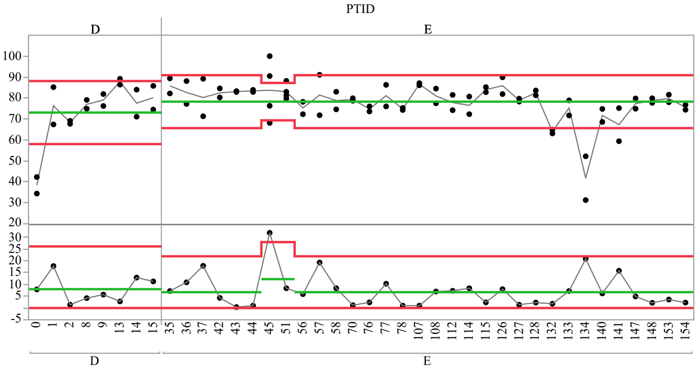

CD14lo Monocytes PDL1 Median

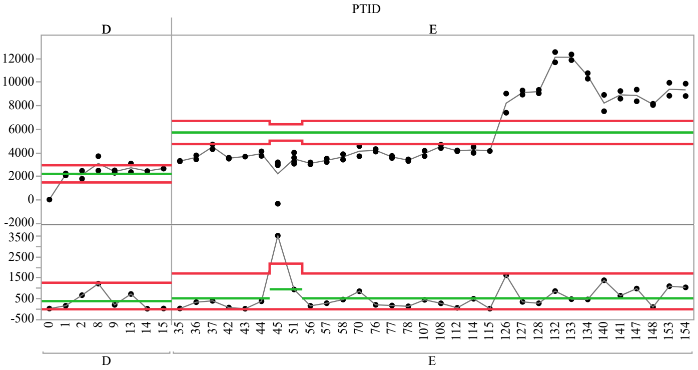

CD4 CM

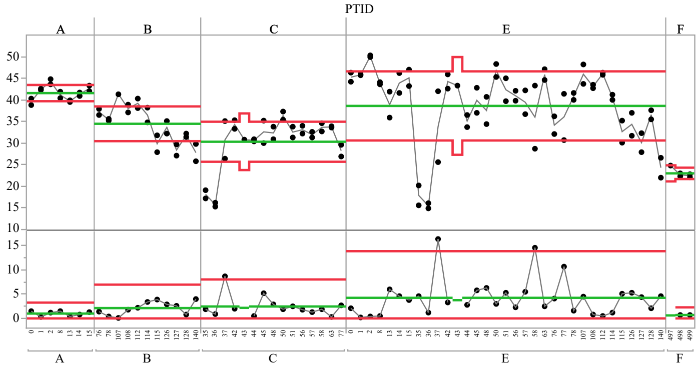

CD4 CXCR3+

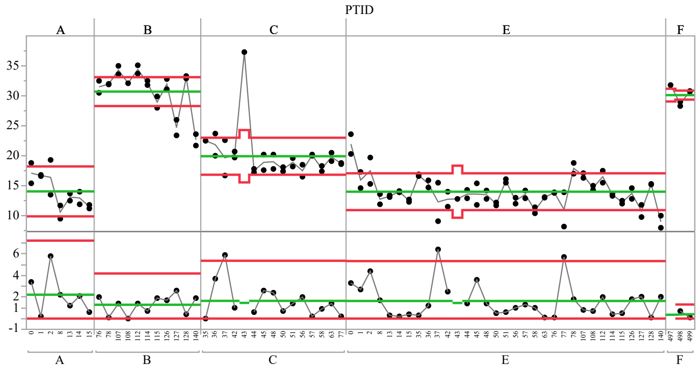

CD4 EM

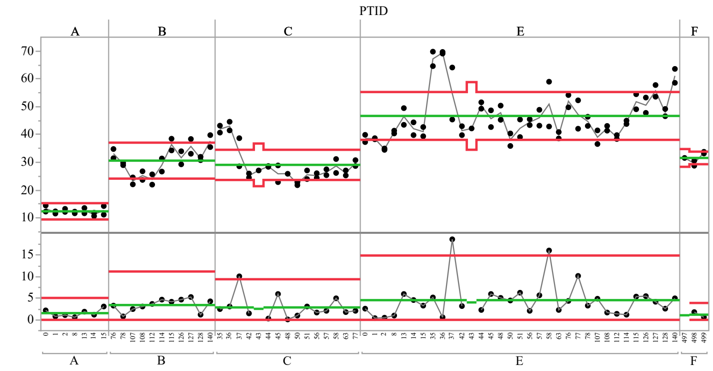

CD4 N

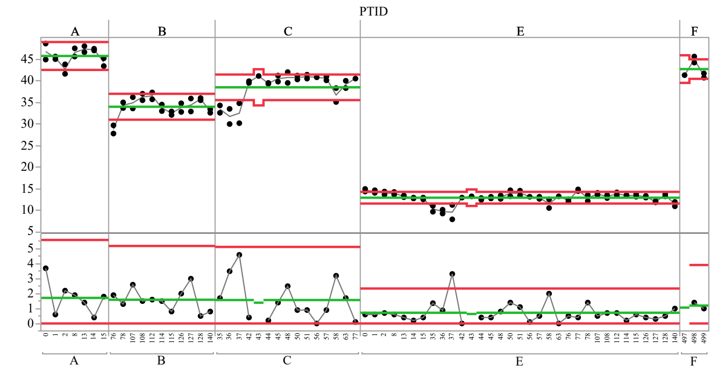

CD4 pool Avg Spot Count

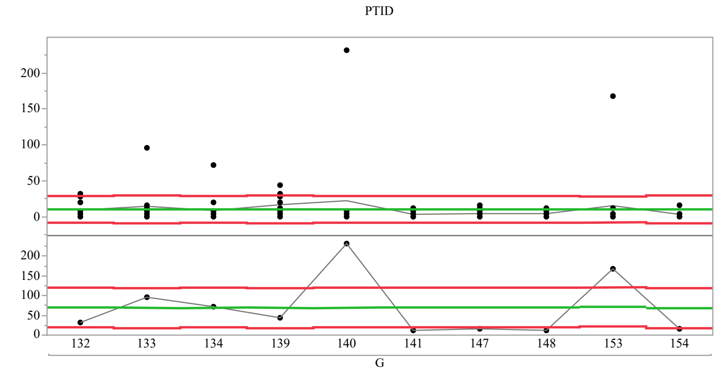

CD4 pool IFNg

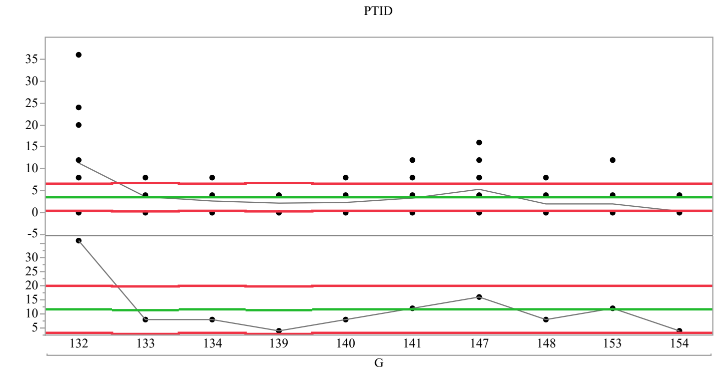

CD4 pool IFNg/IL-2

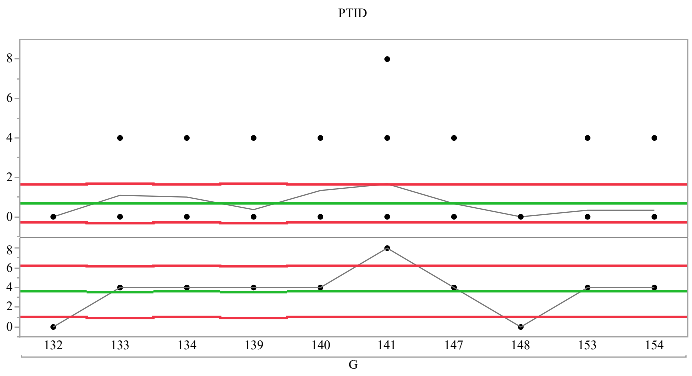

CD4 pool IL-2

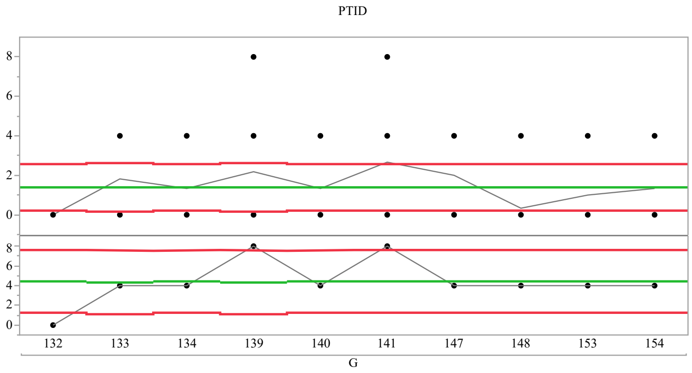

CD4 TEMRA

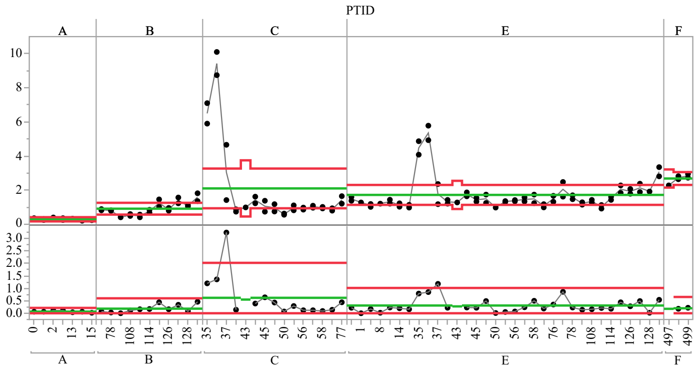

CD8 CM

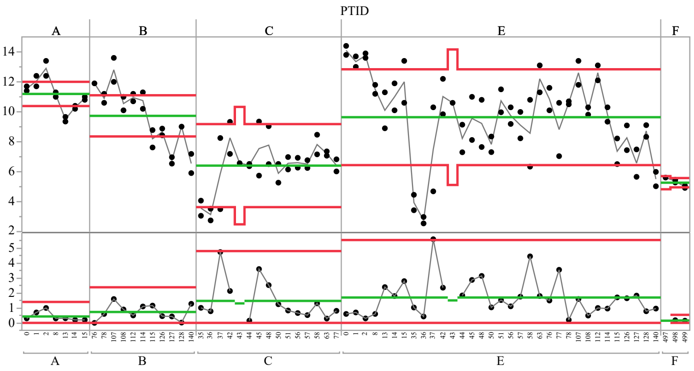

CD8 CXCR3+

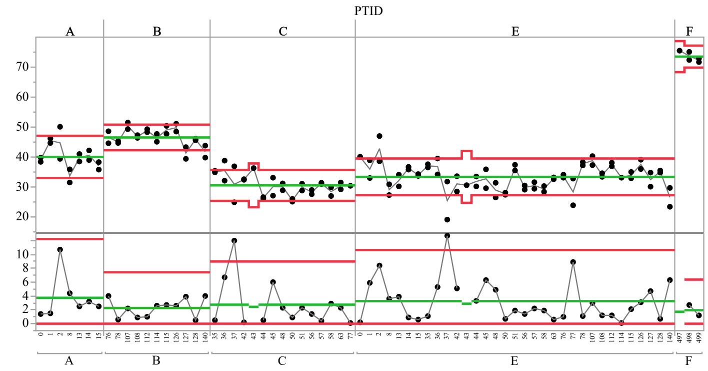

CD8 EM

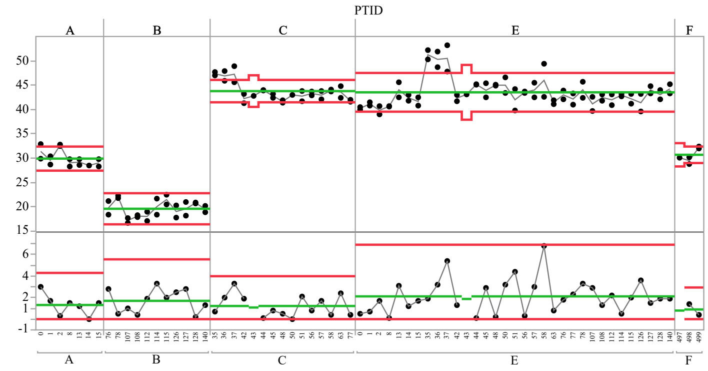

CD8 N

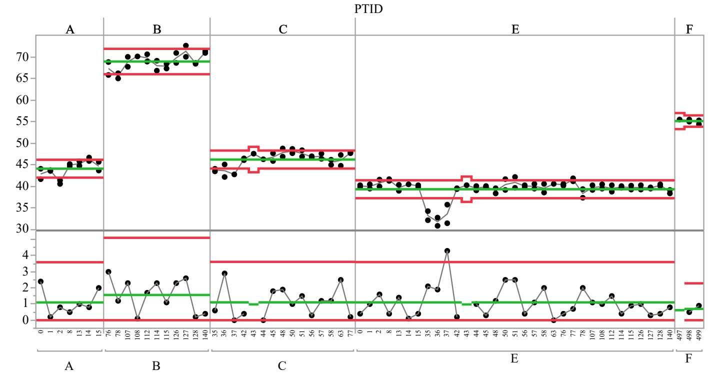

CD8 TEMRA

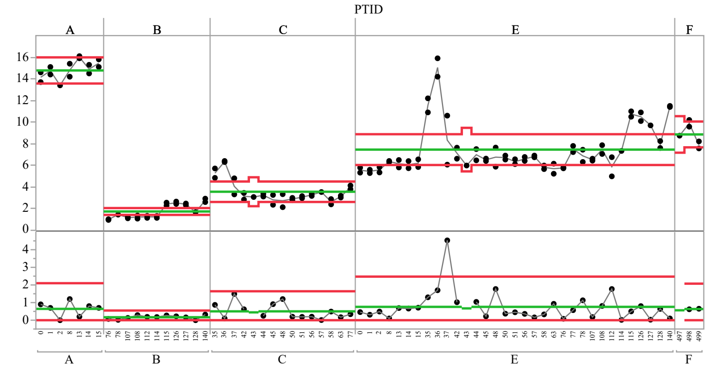

NK Cells

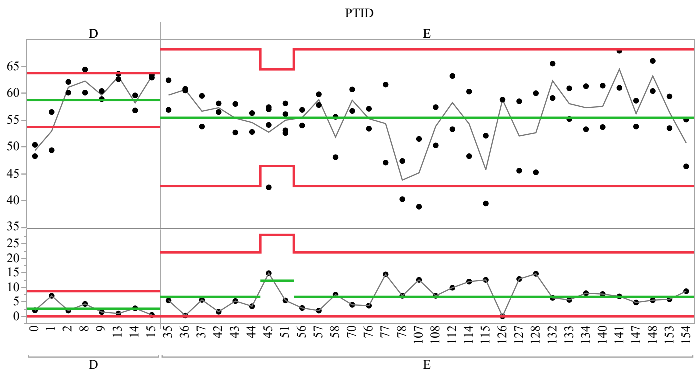

NK Cells CD2+

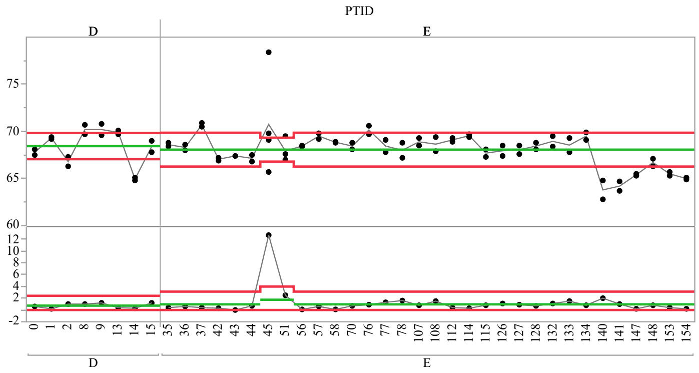

NK Cells CD36+

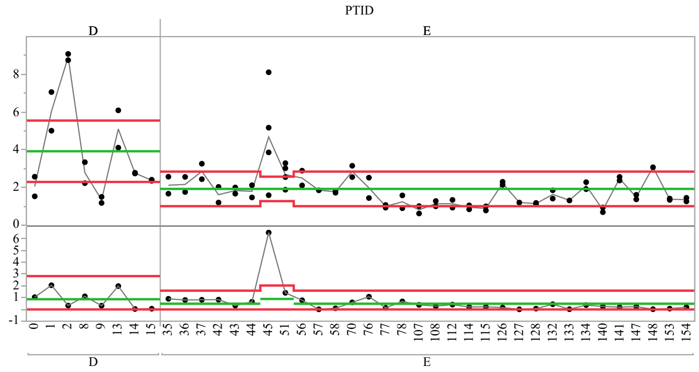

NK Cells CD54+

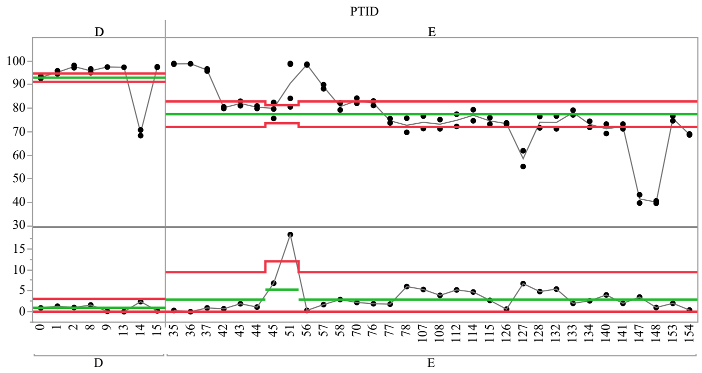

NK Cells CD57+

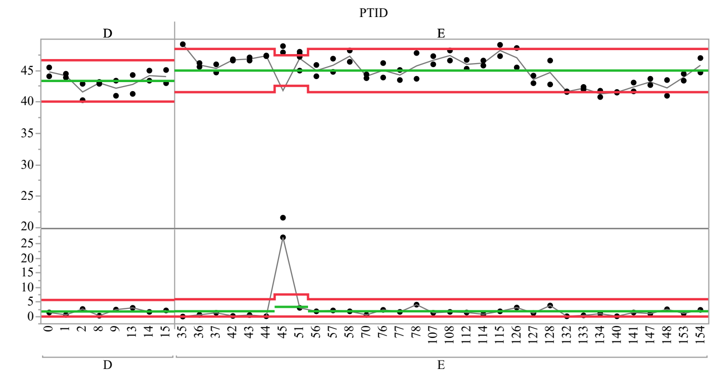

NK Cells NKG2D+

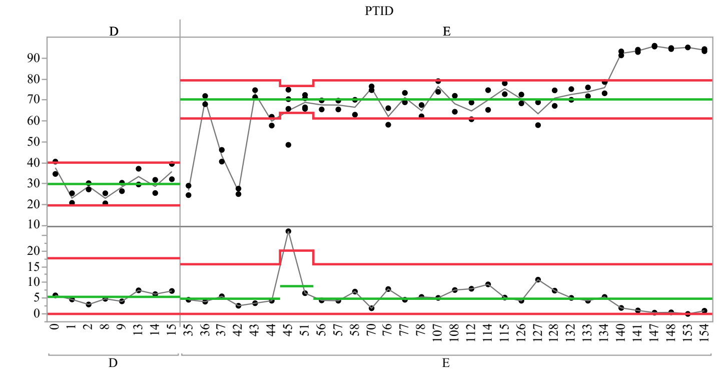

Control Chart Builder Analyte=NK Cells NKp46+

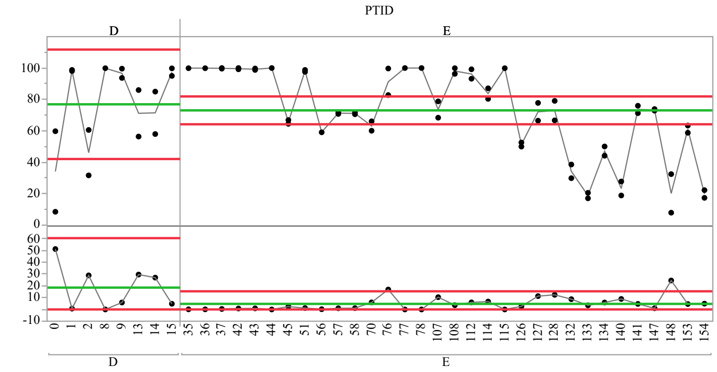

NK Cells PDL1 Median

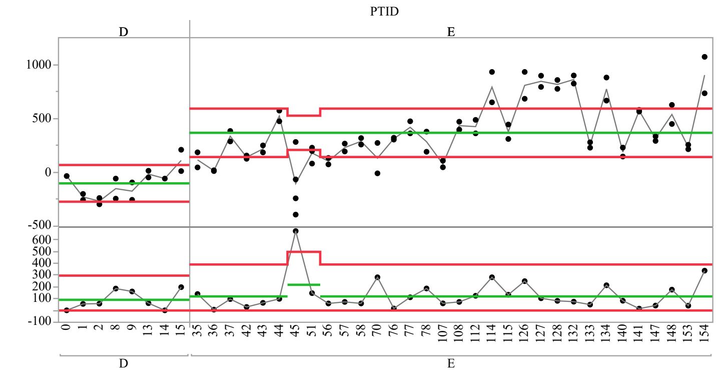

NKhi Cells

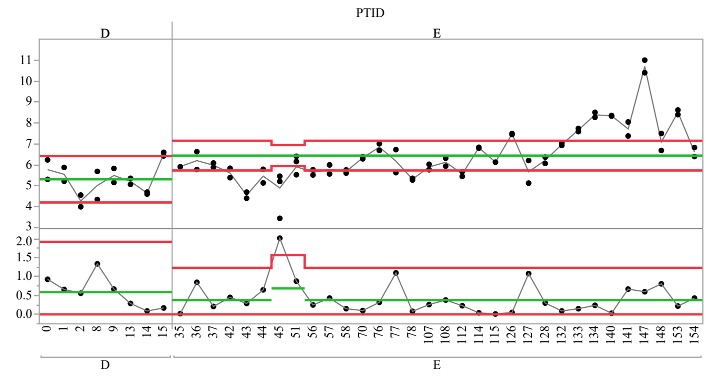

NKhi Cells CD2+

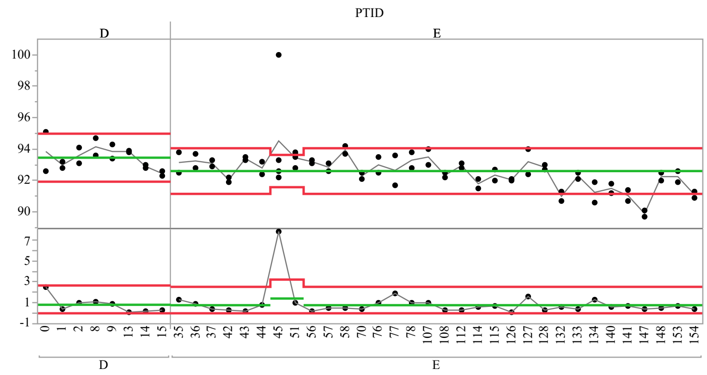

NKhi Cells CD36+

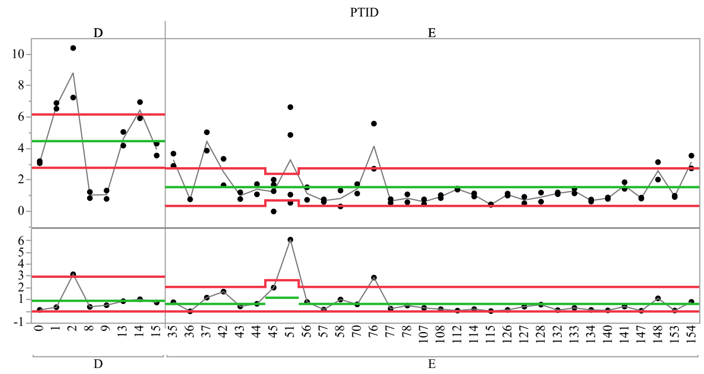

NKhi Cells CD54+

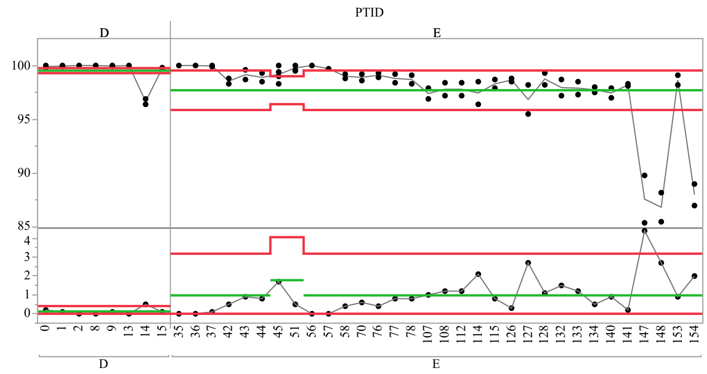

NKhi Cells CD57+

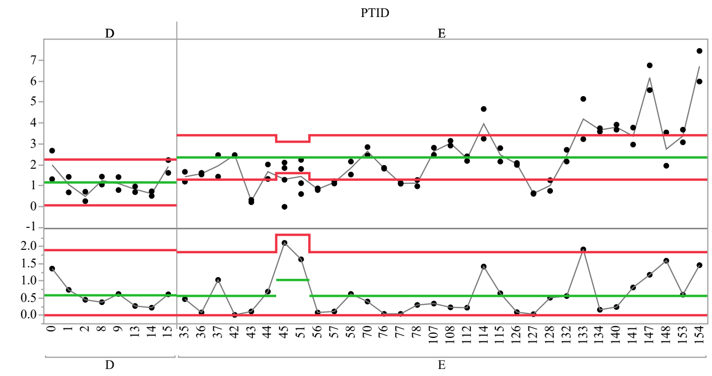

NKhi Cells NKG2D+

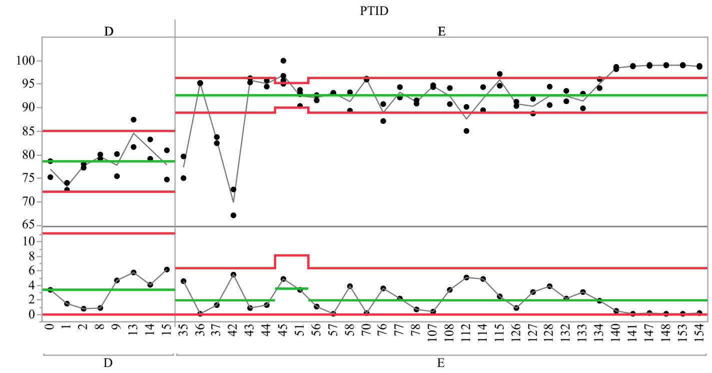

NKhi Cells NKp46+

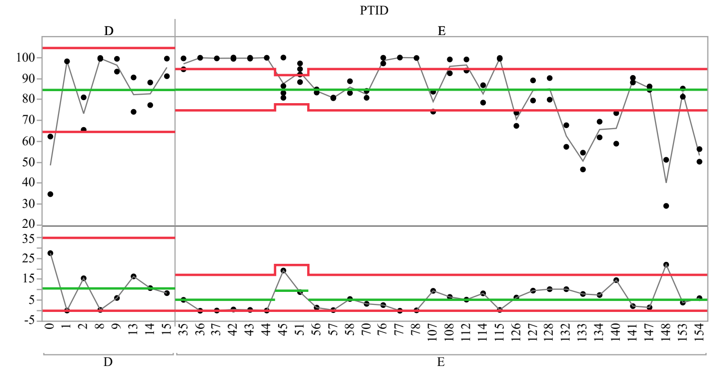

Nkhi Cells PDL1 Median

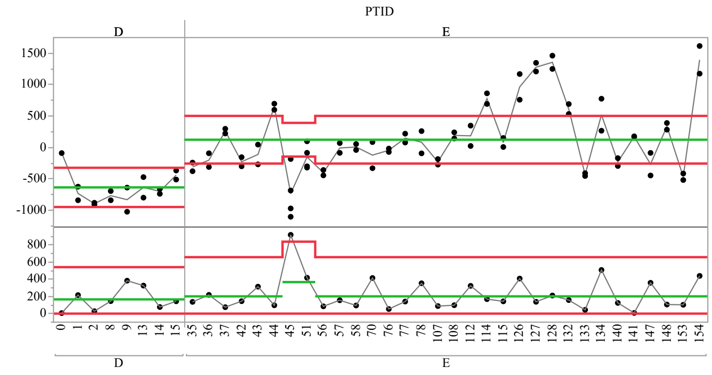

Preproinsulin Avg Spot Count

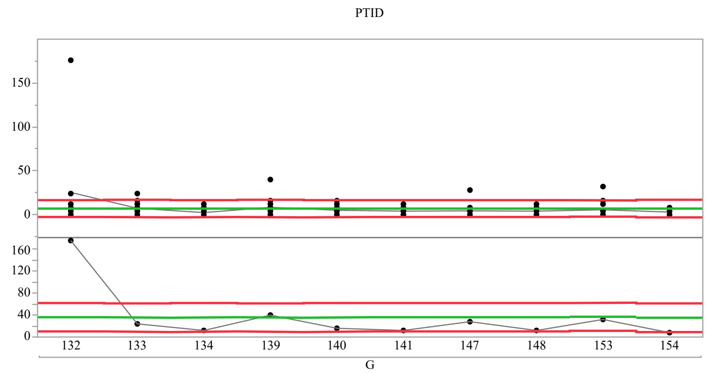

Preproinsulin IFNg

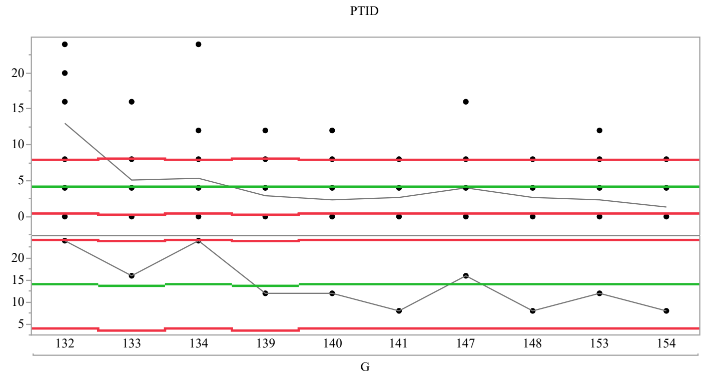

Preproinsulin IFNg/IL-2

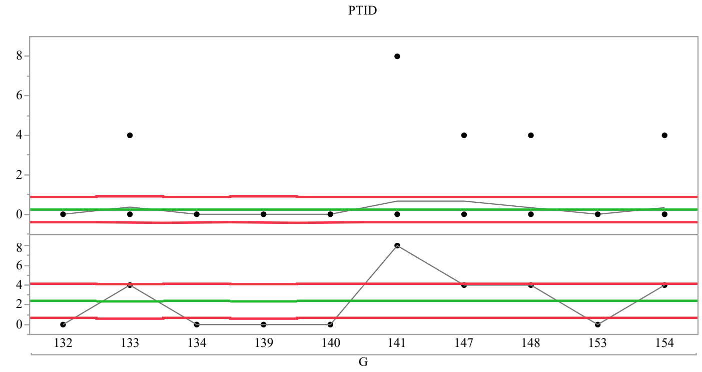

Preproinsulin IL-2

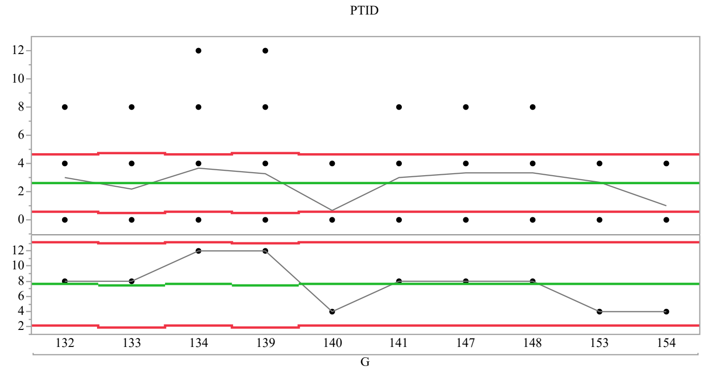

QDM pool Avg Spot Count PTID

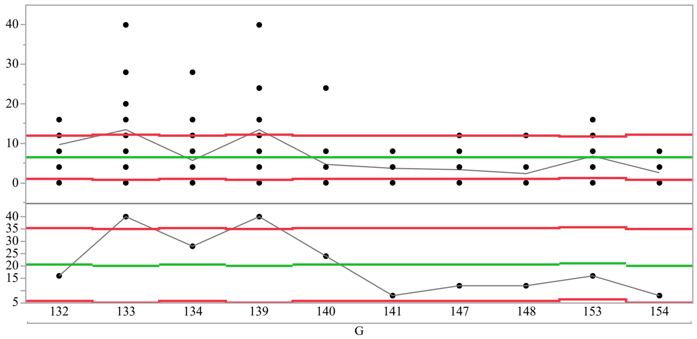

QDM pool IFNg

PTID

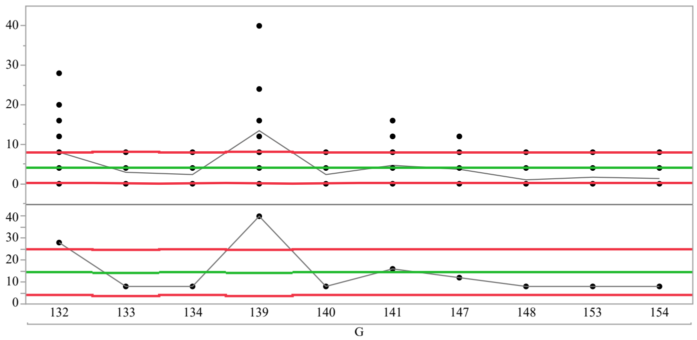

QDM pool IFNg/IL-2

PTID

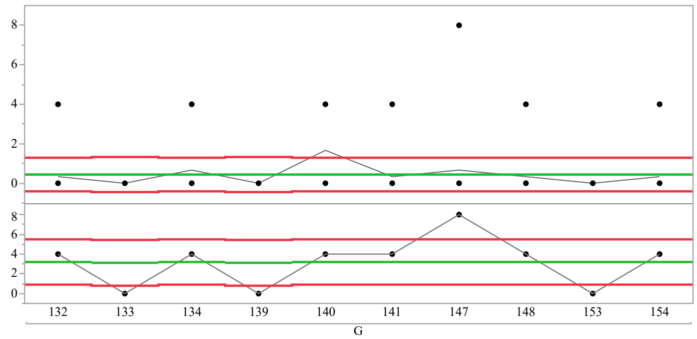

QDM pool IL-2

PTID

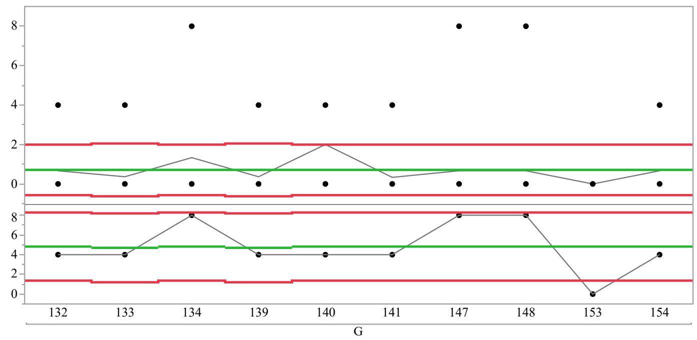

Sum of AgSpc CD8 CM

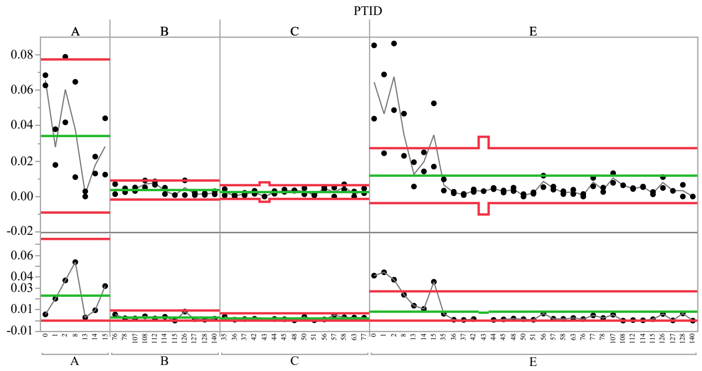

Sum of AgSpc CD8 CM (No INS)

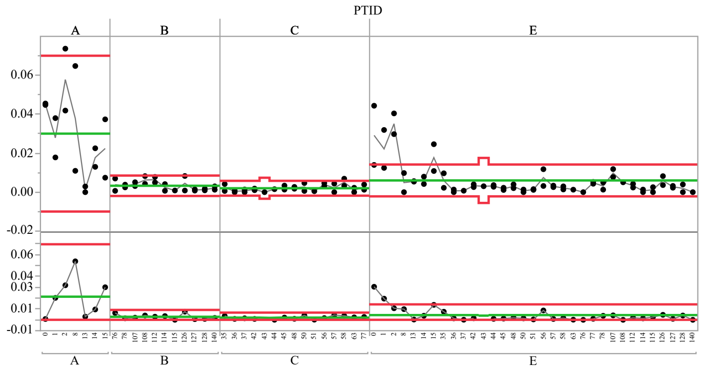

Sum of AgSpc CD8 CM CXCR3+

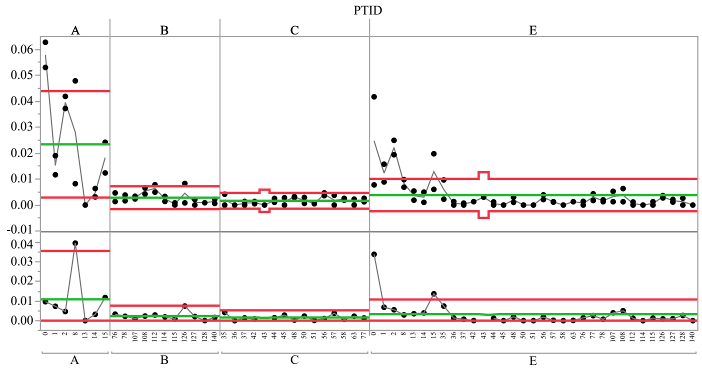

Sum of AgSpc CD8 CM CXCR3+ (No INS)

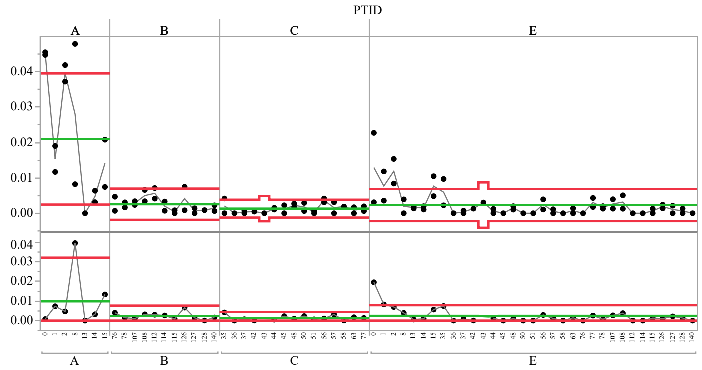

Sum of AgSpc CD8 EM

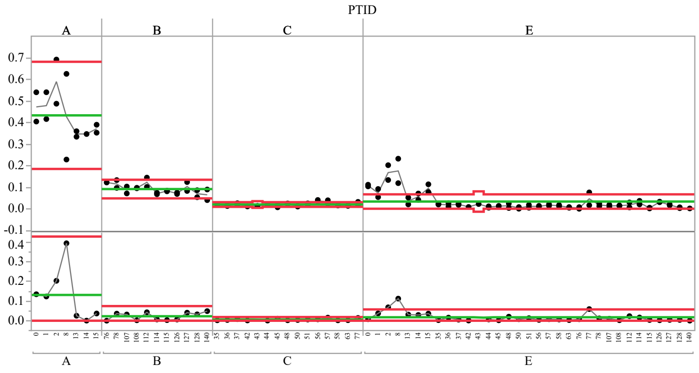

Sum of AgSpc CD8 EM (No INS)

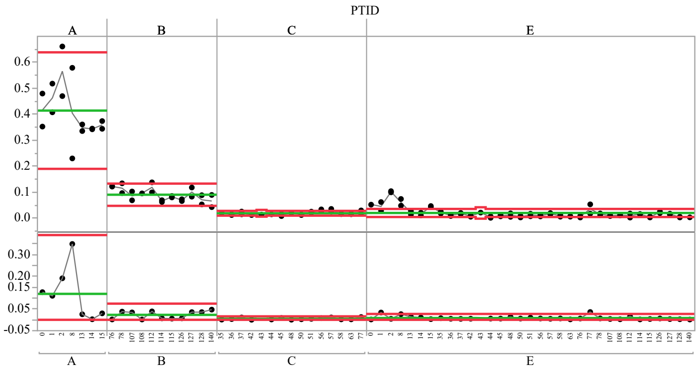

Control Chart Builder Analyte=Sum of AgSpc CD8 EM CXCR3+

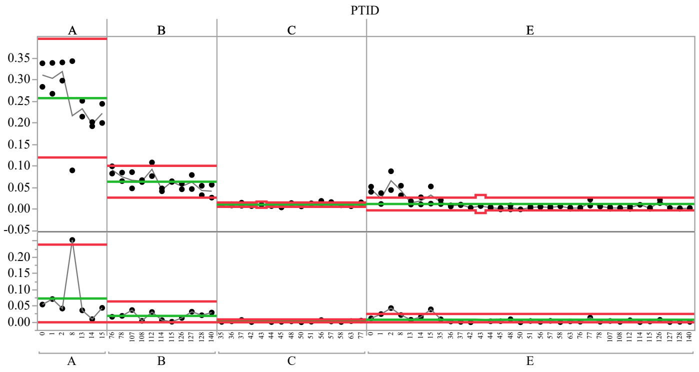

Sum of AgSpc CD8 EM CXCR3+ (No INS)

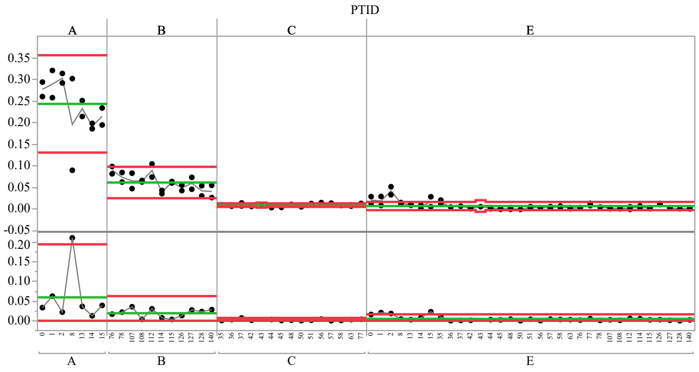

Sum of AgSpc CD8 TEMRA

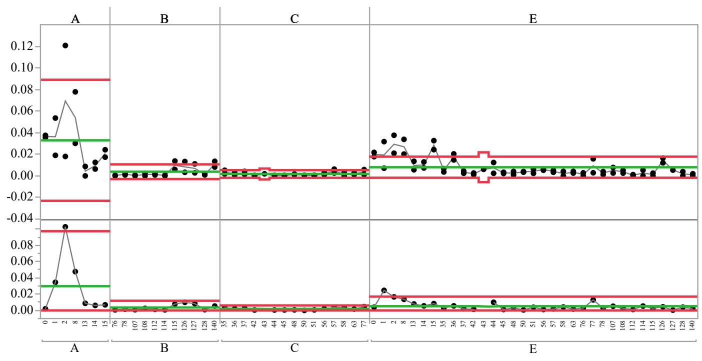

Sum of AgSpc CD8 TEMRA (No INS)

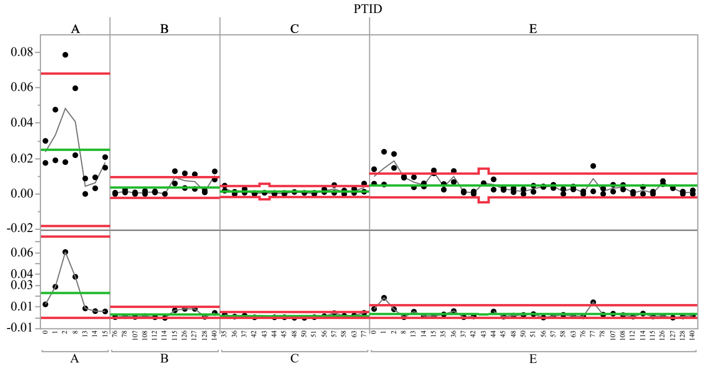

Sum of AgSpc CD8 TEMRA CXCR3+

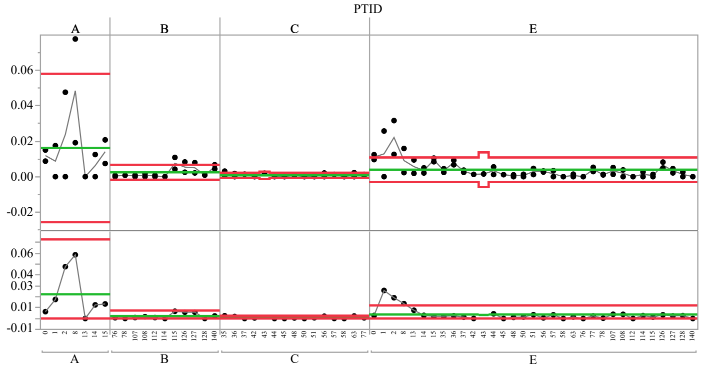

Sum of AgSpc CD8 TEMRA CXCR3+ (No INS)

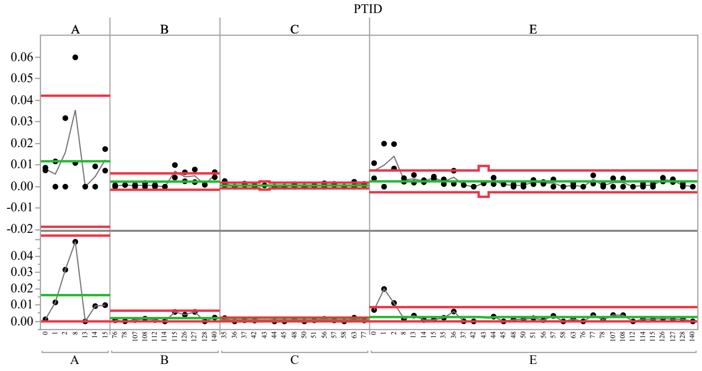

Supplement: Supplementary file 2 [file Data_Sheet_2.pdf]
